# One-dimensional *vs.* two-dimensional proton transport processes at solid–liquid zinc-oxide–water interfaces†
†Electronic supplementary information (ESI) available: Snapshots (Cartesian coordinates of the atoms including periodic lattice parameters) of the ZnO(101[combining macron]0) and ZnO(112[combining macron]0) surfaces in contact with liquid water. Details for the automated PT coordinate assignment. Calculated PTFELs for all proton transfer coordinates, and two-dimensional PTFELs for the PT coordinates in Fig. 4. Validation of the neural network by comparison to *ab initio* molecular dynamics simulations. See DOI: 10.1039/c8sc03033b


**DOI:** 10.1039/c8sc03033b

**Published:** 2018-11-05

**Authors:** Matti Hellström, Vanessa Quaranta, Jörg Behler

**Affiliations:** a Universität Göttingen , Institut für Physikalische Chemie, Theoretische Chemie , Tammannstr. 6 , 37077 Göttingen , Germany . Email: joerg.behler@uni-goettingen.de; b Lehrstuhl für Theoretische Chemie , Ruhr-Universität Bochum , 44780 Bochum , Germany

## Abstract

Long-range charge transport is important for many applications like batteries, fuel cells, sensors, and catalysis. Obtaining microscopic insights into the atomistic mechanism is challenging, in particular if the underlying processes involve protons as the charge carriers. Here, large-scale reactive molecular dynamics simulations employing an efficient density-functional-theory-based neural network potential are used to unravel long-range proton transport mechanisms at solid–liquid interfaces, using the zinc oxide–water interface as a prototypical case. We find that the two most frequently occurring ZnO surface facets, (101[combining macron]0) and (112[combining macron]0), that typically dominate the morphologies of zinc oxide nanowires and nanoparticles, show markedly different proton conduction behaviors along the surface with respect to the number of possible proton transfer mechanisms, the role of the solvent for long-range proton migration, as well as the proton transport dimensionality. Understanding such surface-facet-specific mechanisms is crucial for an informed bottom-up approach for the functionalization and application of advanced oxide materials.

## Abstract 2

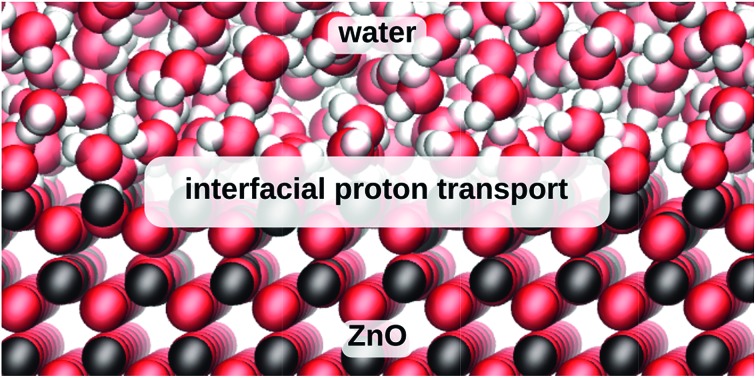
Neural network molecular dynamics simulations unravel the long-range proton transport properties of ZnO–water interfaces.

## Introduction

Proton *transfer* (PT) is the process in which a proton (H^+^) is transferred from one molecule to another. PT reactions play an important role for acid/base chemistry, heterogeneous and homogeneous catalysis, corrosion, biochemistry, and applications like proton exchange membrane fuel cells, where the protons act as charge carriers.

**Fig. 1:**
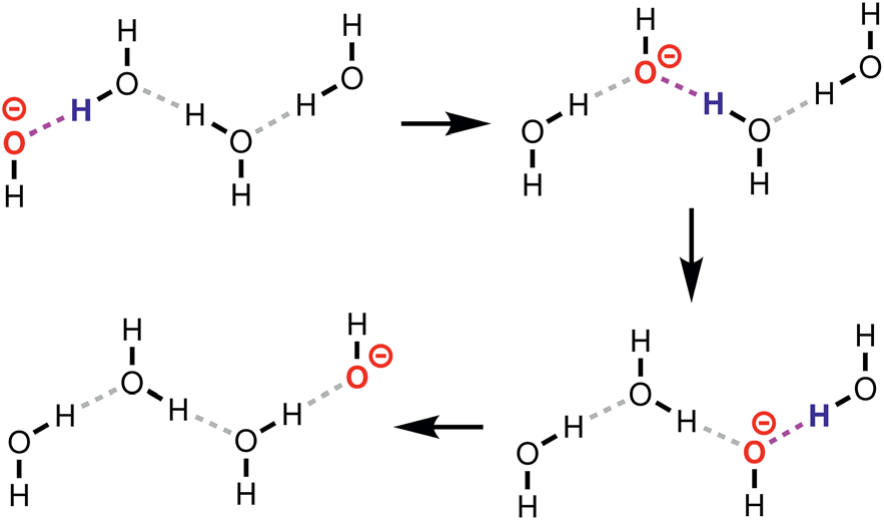
Schematic representation of the Grotthuss mechanism for the transport of OH^–^ (red) in water. The protons that participate in the next reaction step are shown in blue, and the hydrogen bond along which they are transferred in magenta. Only the molecules in the water “wire” along which the proton hole diffuses are shown. Each step in the mechanism is aided by fluctuations in the hydrogen bond network with the surrounding water molecules (not shown).

The term proton *transport* is often used to refer to the long-scale diffusion of protons in a system. In aqueous solutions containing hydronium (H_3_O^+^) and hydroxide (OH^–^) ions, proton transport proceeds *via* the Grotthuss mechanism (reviewed in, for example, ref. 1), in which charge and mass transport are largely decoupled. A schematic representation of this mechanism for OH^–^(aq) is given in Fig. 1, where protons are transferred from water molecules to hydroxide ions. One sometimes uses a different perspective, namely that proton “holes”, *i.e.*, missing protons, are transferred from the hydroxide ion to the water molecule.^2^,^3^ The Grotthuss mechanism then becomes a series of proton hole transfer events. The transport mechanisms of protons and hydroxide ions in aqueous solution have been extensively studied,^2^–^9^ but only little is known about the proton transport mechanisms at solid–liquid interfaces. For instance, recent molecular dynamics (MD) studies^10^,^11^ have shown that OH^–^ diffuses both vehicularly (mass transport) and *via* a Grotthuss-like mechanism in anion exchange membranes. Further, Muñoz-Santiburcio and Marx^3^ explored OH^–^ diffusion in nanoconfined slit pores, and concluded that the transport mechanism depends on the degree of confinement. Achtyl *et al.*^12^ showed that protons can diffuse through hydroxyl-terminated atomic defects in single-layer graphene.

Metal oxides are abundant in the environment and have found many applications in chemistry. On metal oxide surfaces, water molecules often spontaneously dissociate, for example by transferring a proton to the oxide ion of the substrate. The populations of dissociated and molecular water molecules depend on the substrate, the presence or absence of defects, the coverage of water, the pH, and many other factors. Often, a mixed molecular/dissociated water layer forms at the surface.

A typical metal oxide with mixed molecular/dissociated water adsorption is zinc oxide, ZnO. The interface between ZnO and water appears in, for example, medicinal chemistry,^13^ biosensors^14^ as well as pH sensors,^15^ and photocatalysts.^16^–^18^ Zinc oxide nanoparticles can be selectively grown into many different shapes and sizes.^19^ The most common crystal structure of ZnO is hexagonal wurtzite (space group *P*6_3_*mc*), in which each Zn^2+^ and O^2–^ ion is approximately tetrahedrally coordinated. The *c* crystal direction, with Miller indices [0001], is polar. Thus, the [0001] and [0001[combining macron]] crystal directions are not equivalent. The polar ±(0001) surfaces are less stable than the two most stable nonpolar (101[combining macron]0) and (112[combining macron]0) surfaces:^20^,^21^ for this reason, ZnO nanowires are normally longer along the ±[0001] crystal directions, exposing mainly (101[combining macron]0) and/or (112[combining macron]0) surface facets.^19^ The present work addresses proton transfer and proton transport at those two surfaces of ZnO.

The interaction of water with ZnO surfaces has been the subject of numerous theoretical studies, for water coverages ranging from sub-monolayer (sub-ML),^22^–^24^ to one^22^,^23^,^25^–^28^ and a few MLs,^24^,^29^,^30^ to the liquid.^31^–^35^ In general, it has been found that an increase of the water coverage leads to a greater degree of water dissociation at the surface. Water molecules adsorbed on surface Zn ions (Zn_s_^2+^), here denoted O*H_2_, can dissociate and recombine by transferring a proton to/from a nearby surface oxide ion (O_s_^2–^):

1 O_s_H^–^ + O*H^–^ ⇌ O_s_^2–^ + O*H_2_

where we have arbitrarily chosen to have the forward reaction correspond to water recombination, and the backward reaction correspond to water dissociation. Above, we consider the proton adsorbed on the oxide ion to form a “surface hydroxide ion” (O_s_H^–^). In a previous work,^34^ we called the above reaction “surface-PT”, because of the participation of the surface oxide ion. At ZnO(101[combining macron]0), numerous studies^32^–^34^ have indicated that PT can also happen between an adsorbed hydroxide ion (O*H^–^) and an adsorbed water molecule (“adlayer-PT”):

2 O*H_2_ + O*H^–^ ⇌ O*H^–^ + O*H_2_

Our previous work^34^ investigated the above two types of PT mechanisms at the ZnO(101[combining macron]0)–liquid-water interface using MD simulations. We found that at ZnO(101[combining macron]0), the rate of adlayer-PT is greater than the rate of surface-PT. Moreover, we and others^33^ found that the PT reactions are aided by hydrogen-bond fluctuations in the immediate environment around the dissociating water molecules, similar to PT reactions in NaOH solutions of high concentrations.^36^

On other metal oxides, such as TiO_2_,^37^ ZrO_2_,^38^ Fe_2_O_3_ (ref. 39) and CeO_2_,^40^ and other materials like GaN,^41^ InP,^42^ and GaP,^42^ it has been demonstrated that also solvent water molecules, *i.e.*, water molecules that are not directly adsorbed on the surface, can participate in proton transfer reactions near the interface. In this work, we will show that such solvent-assisted PT reactions are possible also at the ZnO–water interface.

Although the mechanisms governing *single* PT events have been extensively studied at a number of metal oxides, much less is known about how, and to what extent, *multiple* subsequent PT events at the solid–liquid interface collectively contribute to long-range proton transport or proton *diffusion* as we will call it from now on. The distinction between single and multiple events is important, as multiple proton transfer events do not necessarily lead to any proton diffusion, since protons can jump back and forth, or “rattle”, between the same pair of donors/acceptors multiple times. Nevertheless, proton diffusion *via* the Grotthuss mechanism consists of a series of concatenated PT events. To what extent the short-range local structure, crystalline long-range order, and structural anisotropies of metal oxide surfaces determine the possible pathways for proton transport at the solid–liquid interface, is still completely unknown. Here, we will address these points by obtaining atomic-level insights into the structure and dynamics of the ZnO–liquid-water interface using molecular dynamics simulations.

Proton diffusion at the metal-oxide–liquid-water interface is a challenging case for molecular simulation methods, since the underlying framework must be capable of describing with high accuracy an ionic crystal, a molecular liquid, the interface between them, as well as proton transfer events. Moreover, in order to minimize the influence of finite-size effects, which can be particularly pronounced for diffusion phenomena,^43^ a large system, both with respect to the area of the interface, as well as the thickness of the liquid phase, is needed. At the same time, although individual proton transfer events can be quite fast, long-scale proton diffusion is potentially much slower, thus requiring long trajectories. For these reasons, a computationally efficient method is needed. In this work, we use a reactive density-functional-theory-based high-dimensional neural network potential,^44^,^45^ which provides a computationally inexpensive way of evaluating the total energy and atomic forces in a system maintaining first principles accuracy. Neural networks, one of the most widely used machine learning techniques, are in many ways ideal for simulating complicated processes like proton diffusion at solid–liquid interfaces, since they can be parameterized to reproduce density-functional-theory-calculated potential energy surfaces of arbitrary systems very accurately at a fraction of the computational cost:^44^,^45^ in a previous work, we created and validated such a neural network potential for ZnO–liquid-water interfaces.^34^ The training and validation sets included ample numbers of structures for both the ZnO(101[combining macron]0) and ZnO(112[combining macron]0) surfaces in contact with liquid water. Additional validation of the neural network was performed by comparing, for example, proton transfer free energy landscapes as calculated by the neural network MD simulations to landscapes calculated directly from *ab initio* MD simulations for small systems (see ref. 34, as well as the ESI† to the present work). The comparison to *ab initio* MD revealed satisfactory agreement between the NN and the DFT results, although the NN proton transfer barriers were somewhat underestimated compared to the DFT reference. Moreover, in ref. 34 we highlighted that running a short 25 ps trajectory was not enough for equilibrating the proton transfer free energy landscapes, thus further justifying the need for an atomistic potential (in our case a neural network potential) that can be used to tackle large length and time scales.

**Fig. 2:**
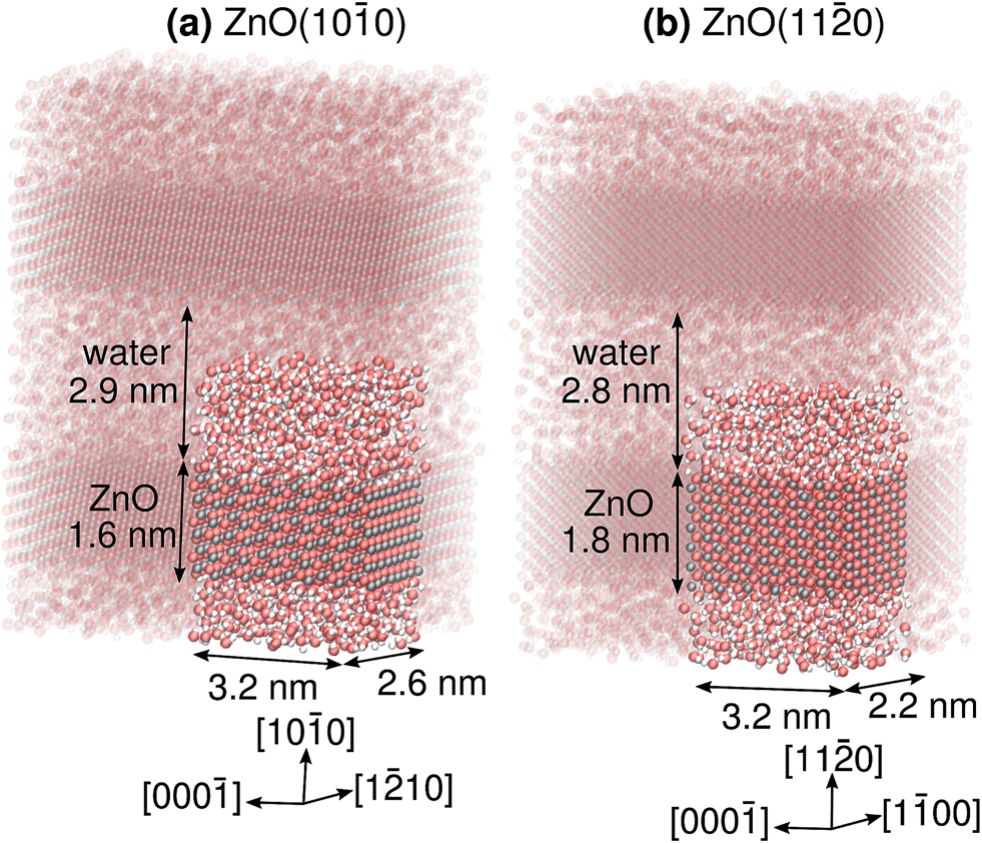
Snapshots from molecular dynamics simulations of (a) the ZnO(101[combining macron]0), and (b) the ZnO(112[combining macron]0) surface in contact with liquid water, illustrating the slab models used in this work. The simulations were performed under three-dimensional periodic boundary conditions. One periodic image for each system, with the given dimensions, is highlighted.

Here, we investigate proton transfer and proton diffusion at the two most prevalent surface facets of ZnO particles, namely ZnO(101[combining macron]0) and ZnO(112[combining macron]0), in contact with a thick liquid water film. The slab models for these two systems are shown in Fig. 2. In both cases, the polar ±[0001] directions run parallel to the surface. We will for the first time decipher (i) differences and similarities between the individual PT mechanisms (*short-range* proton transfer) and how they relate to the structures of the two surfaces, (ii) to what extent the PT barriers along the polar [0001] and [0001[combining macron]] directions differ, and (iii) whether there are preferred proton diffusion directions along the surface. We will demonstrate that proton diffusion, *i.e.*, *long-range* proton transport, on ZnO(101[combining macron]0) is “pseudo-one-dimensional” with hardly any diffusion along the polar crystal directions, whereas it is two-dimensional on ZnO(112[combining macron]0), with significant diffusion along both the polar and non-polar crystal directions.

## Results

The ZnO(101[combining macron]0) and ZnO(112[combining macron]0) surfaces are mixed-terminated, meaning that they expose an equal amount of Zn^2+^ and O^2–^ ions in the outermost surface layer. As compared to the atoms in the bulk, the atoms in the surface layer have each lost one of their four nearest neighbors. For ZnO(101[combining macron]0), only one of the remaining three nearest neighbors is in the surface layer (the remaining two are in the subsurface layer), whereas for ZnO(112[combining macron]0), two of the nearest neighbors are in the surface layer (and one in the subsurface layer). Thus, the ZnO(101[combining macron]0) surface consists of an array of isolated Zn–O “surface dimers” in which two surface Zn atoms are only connected *via* a nearest neighbor O in the subsurface layer. On the contrary, the ZnO(112[combining macron]0) surface consists of extended zig-zagged “surface rows” that consist of nearest-neighbors within in the surface layer; these surface rows extend along the polar ±[0001] directions. The rows are separated by trenches and connected by atoms in the subsurface layer.

Similar to the perspective of “proton hole” diffusion for exploring the Grotthuss mechanism of OH^–^ diffusion in water, we here adopt the convention of exploring “proton hole diffusion” at the ZnO–water interface. To this end, we define “proton hole centers” (PHCs) to be typical proton acceptors, *i.e.* “free” (unprotonated) surface oxide ions, O_s_^2–^, as well as the combination of adsorbed hydroxide ions (O*H^–^) and the corresponding surface Zn ion. We use the term proton hole in this context simply as an indicator for a “missing” proton; our use of this term does not imply that the proton hole diffusion mechanism on the surface, which will be explored below, is equivalent to the archetypal proton hole diffusion mechanism for OH^–^ in water (for a full account of the differences between *proton* and *proton hole* diffusion mechanisms in water, see ref. 1). In our analysis, we could in principle have explored proton diffusion instead, but this would have led to rather complicated definitions of “proton centers” (see ESI†).

**Fig. 3:**
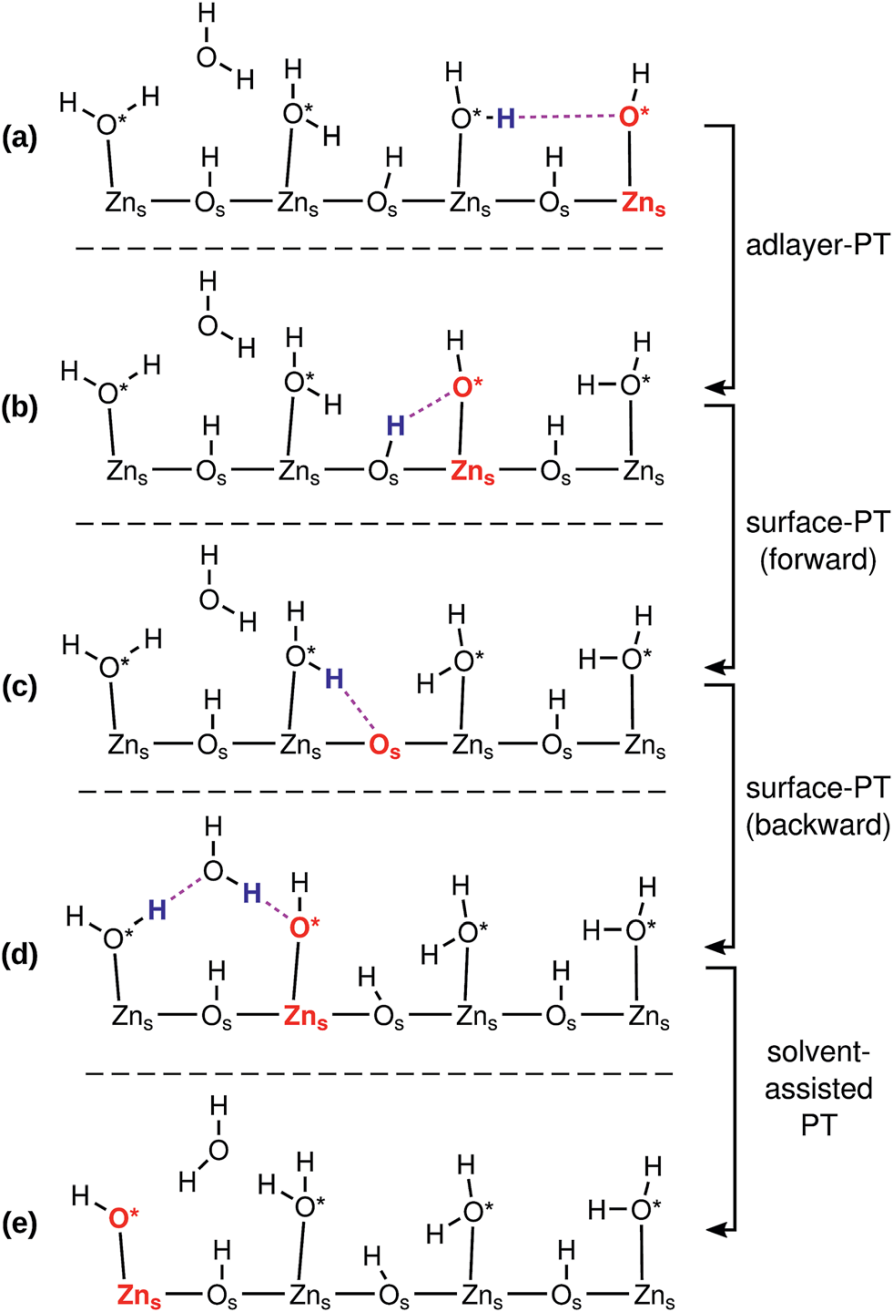
Schematic illustration of the diffusion of a proton hole center (PHC, marked in red; either O*H^–^ adsorbed on Zn_s_^2+^, or a “free” O_s_^2–^) *via* a series of proton transfer (PT) events. The protons that participate in the next PT event are marked in blue, and the hydrogen bond along which they are transferred in magenta. Charges have been omitted for clarity. The figure shows only a schematic, general, representation of a mixed-terminated ZnO surface. The actual structure of the surface influences the possible sequence and rate of PT reactions. The liquid water film is not shown in the figure, although it is present in the simulations.

Fig. 3 indicates how a PHC (red) can diffuse on a ZnO surface *via* a series of PT events. The ZnO surface is depicted only schematically; the true two and three-dimensional structures of the surfaces influence the possible sequence of PT events, and the directions in which the PHC diffuses, as we will explore in more detail below.

Fig. 3 illustrates the four different kinds of PT events that occur at the interface: adlayer-PT (eqn (2)), forward surface-PT (where a water molecule recombines, eqn (1)), backward surface-PT (where a water molecule dissociates, eqn (1)), as well as solvent-assisted PT. Fig. 3 marks both the O in O*H^–^ as well as the Zn_s_^2+^ on which it is adsorbed as constituting the “proton hole center”. Both viewpoints have advantages, and we will switch between them when convenient. Associating the Zn_s_^2+^ with the PHC will give us a better overview of the network of proton diffusion pathways on the surface. The surface-PT reaction in eqn (1) can thus also be written as (*cf.*Fig. 3b–d)

3 PHC(Zn_s_^2+^) ⇌ PHC(O_s_^2–^).

Similarly, the adlayer-PT (eqn (2)) and solvent-assisted PT reactions can be written as

4 PHC(Zn_s_^2+^{1}) ⇌ PHC(Zn_s_^2+^{2})

where {1} and {2} indicate that the PHC is transferred from one Zn_s_^2+^ to another.

Surface-PT events affect the degree of surface hydroxylation *α*, which we define as the fraction of surface oxygen species that form a surface hydroxide, O_s_H^–^, *i.e.*, 
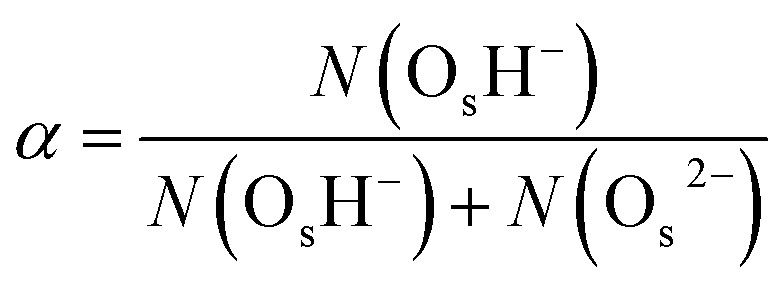
, where *N*(·) is the number of species. We find that the ZnO(112[combining macron]0) surface has a larger degree of hydroxylation (*α* = 0.764 ± 0.009; the error bar indicates the 95% confidence interval around the mean after block-averaging the hydroxylation level over 20 equal-sized portions of the trajectory) than the ZnO(101[combining macron]0) surface (*α* = 0.706 ± 0.002); a greater hydroxylation level at the ZnO(112[combining macron]0)–liquid-water interface was also observed in a previous molecular dynamics study employing a reactive force field.^31^ For ZnO(101[combining macron]0), *ab initio* MD simulations^32^,^33^ have predicted a smaller equilibrium hydroxylation level (*α* ≈ 0.5) than what the neural network potential predicts, which we have previously explained as arising from the different density functionals employed in those simulations compared to ours (with our present neural network potential being parameterized to RPBE-D3 reference data, see also Methods).^34^

**Fig. 4:**
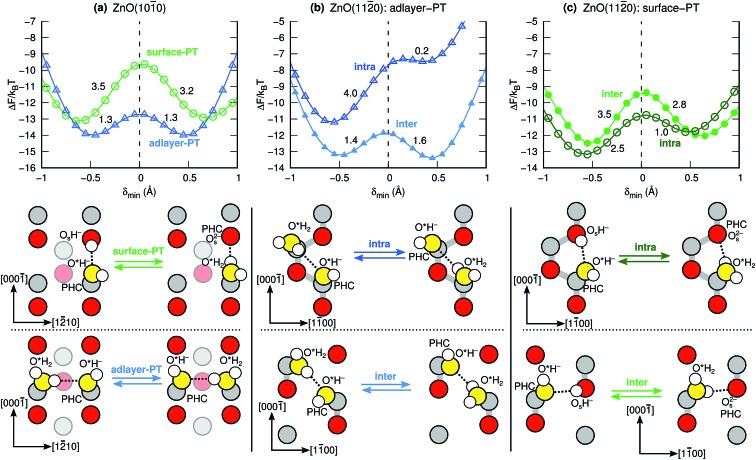
Calculated proton transfer free-energy landscapes (PTFELs) at *T* = 300 K and schematic structures for the surface-PT and adlayer-PT coordinates at the (a) ZnO(101[combining macron]0) and (b and c) ZnO(112[combining macron]0) interfaces in the presence of liquid water (only the species participating in PT are shown). The numbers given in the PTFELs are the corresponding PT barriers in units of *k*_B_*T*. For ZnO(112[combining macron]0) (b and c), proton hole centers (PHCs) are transferred within the same surface “row” (intra), or between two adjacent surface rows (inter); the gray lines connect atoms within the same surface row. In (b), the left and right hand sides of the adlayer-PT coordinates are different because of the polar crystal direction in the substrate.

Fig. 4 shows the calculated proton transfer free-energy landscapes (PTFELs) for the surface-PT and adlayer-PT coordinates at the ZnO(101[combining macron]0) and ZnO(112[combining macron]0) surfaces. Further, top views of the surfaces with only the species participating in the PT reaction are given. The PTFELs give the free-energy barriers Δ*F*^‡^ for transferring the proton from the donor to the acceptor, given that the corresponding donor–acceptor pair has already formed and that the proton is deemed active for PT (see Methods). The PTFEL is presented as a one-dimensional function of the PT coordinate *δ*_min_, which is the difference between the covalent and noncovalent O–H distances; this geometric viewpoint is sufficient for our present purposes. However, the PTFEL is a many-body function, and other relevant coordinates that can be used to characterize it are, for example, the O–O distances,^33^ the number of hydrogen bonds that are donated and accepted by the participating species,^34^,^36^ and the hydrogen-bonding distances to molecules that are *not* involved in the PT itself.^33^ Using both *δ*_min_ and the O–O distances as coordinates, ESI Fig. 6 and 7† show that the PT barriers are smaller for shorter O–O distances, as previously shown also for many other PT reactions.^1^,^33^

The value of the relative free energy Δ*F* at the maximum of the PTFEL can be interpreted as a measure of the rate of PT along a particular PT coordinate.^36^ It is not a measure of the absolute rate (the number of PT events per time unit), but rather, a measure of the relative rate of different PT mechanisms in the same MD trajectory. The assumption is that for a PT event to happen, the PT coordinate *δ*_min_ must pass through the value where Δ*F* is the highest (at *δ*_min_ ≈ 0 Å). A greater value of the free-energy maximum implies a lower PT rate along that particular coordinate. Although the PT barriers can be different for the forward and backward reactions of any PT mechanism, the rates for the forward and backward reactions are equal because of the different population of these states, since the system is in chemical equilibrium.

Fig. 4a shows the surface-PT and adlayer-PT free energies at ZnO(101[combining macron]0). For a full discussion of those results, we refer the reader to our previous work.^34^ Here, we simply note, that for ZnO(101[combining macron]0): (i) the rate of adlayer-PT is greater than the rate of surface-PT, (ii) the left and right hand sides (LHS and RHS) of the adlayer-PT coordinate are equivalent, and (iii) for surface-PT, the LHS [dissociated water, PHC(Zn_s_^2+^)] is more stable than the RHS [molecular water, PHC(O_s_^2–^)].

At the other nonpolar surface, ZnO(112[combining macron]0) (see Fig. 4b–c), there is greater variety of the possible PT coordinates. For both surface-PT and adlayer-PT, the PHC can be transferred between surface atoms that lie within the same surface row (intra-surface-PT and intra-adlayer-PT), or between surface atoms that lie on different rows (inter-surface-PT and inter-adlayer-PT). The greatest PT rate is obtained for the inter-adlayer-PT coordinate, followed by intra-surface-PT, inter-surface-PT, and finally intra-adlayer-PT.

Unlike what was the case at ZnO(101[combining macron]0) (Fig. 4a), the LHS and RHS of the two adlayer-PT coordinates at ZnO(112[combining macron]0) (Fig. 4b) are not equivalent. This is because the direction of adlayer-PT at ZnO(112[combining macron]0) has a component along the polar [0001] direction of the crystal substrate. The PT barriers for the relatively rare intra-adlayer-PT coordinate depend very strongly on whether the proton is transferred along [0001] or [0001[combining macron]], while for the more common inter-adlayer-PT coordinate, the direction of PT has less of an influence (amounting to only about 0.2*k*_B_*T*) on the PT barriers.

**Fig. 5:**
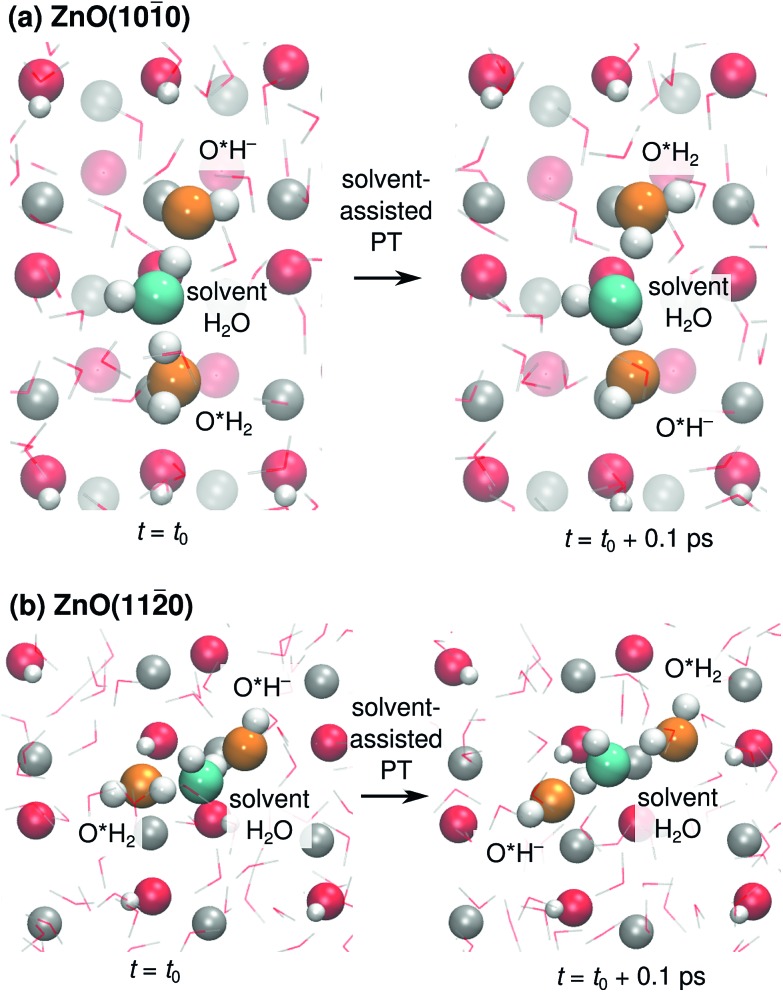
Top views of snapshots illustrating solvent-assisted proton transfer mechanisms at (a) ZnO(101[combining macron]0), and (b) ZnO(112[combining macron]0). The participating solvent water molecule is colored in cyan, the net proton donors and acceptors in orange, and the substrate O and Zn atoms in red and gray, respectively. For ZnO(101[combining macron]0), the subsurface layer is shown grayed-out. Surrounding water molecules are shown as thin lines.

We also found cases of the “solvent-assisted” proton transfer schematically indicated in Fig. 3d–e. Fig. 5 shows actual snapshots from the MD trajectories for this type of PT. Unlike the PT mechanisms in Fig. 4, which involved the transfer of only a single proton, the solvent-assisted PT involves the concerted transfer of *two* protons: one proton is donated *to* the solvent H_2_O by an adsorbed O*H_2_, and one proton is donated *by* the solvent H_2_O to an adsorbed O*H^–^. Solvent-assisted PT proceeds *via* transiently formed solvent OH^–^ and OH_3_^+^ species, that are not stable intermediates (ESI Fig. 3 and 5†). The events are quite rare. For example, in the 44 ns long simulation for ZnO(101[combining macron]0), only 41 solvent-assisted PT events were observed.

**Fig. 6:**
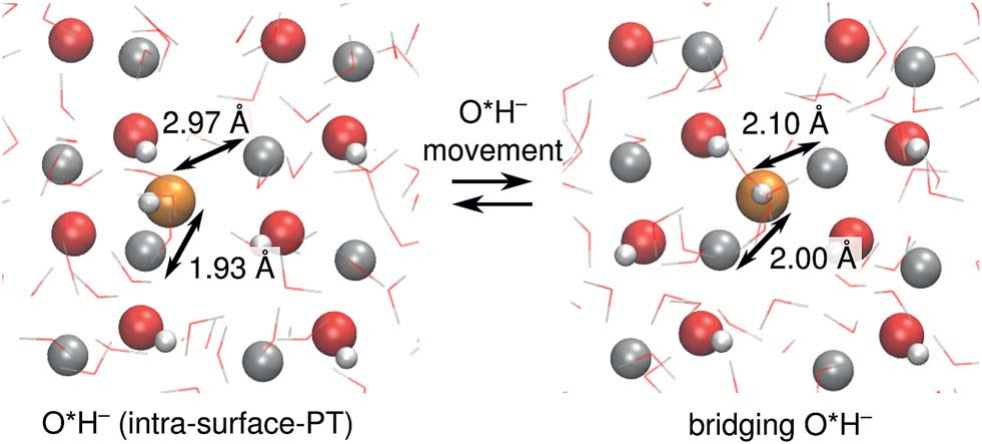
Snapshots of the ZnO(112[combining macron]0) surface illustrating the vehicular movement of O*H^–^ (orange) from the intra-surface-PT coordinate (left) to a configuration where it bridges two adjacent surface rows (right). The instantaneous distances between the O in O*H^–^ and the two nearest Zn_s_^2+^ are given.

At ZnO(112[combining macron]0), O*H^–^ in the intra-surface-PT coordinate (Fig. 4c) can move towards the nearest Zn_s_^2+^ in the neighboring surface row, ending up in a configuration where the O*H^–^ bridges two surface rows (see Fig. 6). Although such bridging species accept a hydrogen bond from O_s_H^–^, they are mostly inactive for PT, as the PT reaction from a bridging O*H_2_ is barrierless (ESI Fig. 4†). Bridging O*H^–^ thus disappear by moving back along [0001] to the intra-surface-PT configuration. About 12% of O*H^–^ at ZnO(112[combining macron]0) are in such a bridging configuration. In the coming analysis, we assign the location of the PHC for such bridging configurations to be the location of the O*H^–^.

**Fig. 7:**
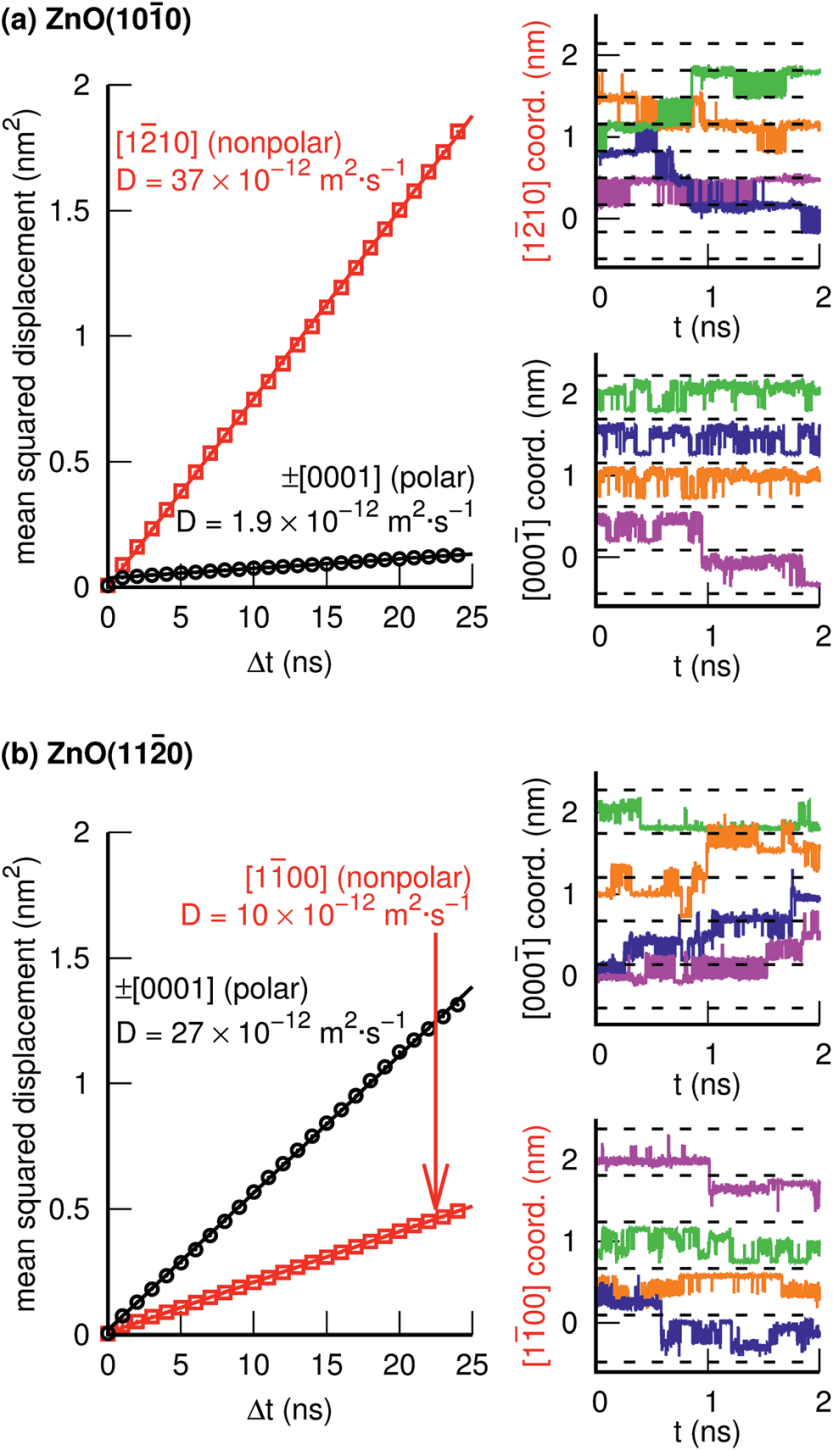
One-dimensional mean squared displacement (MSD) and four example trajectories along two different crystal direction for the PHCs at the (a) ZnO(101[combining macron]0), and (b) ZnO(112[combining macron]0) surfaces. The dashed lines in the trajectory plots are separated by distances equal to the surface unit cell along the corresponding crystal direction.

Fig. 4 and 5 illustrate individual PT events, but give no information about if such PT events can be concatenated to yield Grotthuss-like diffusion of the PHC along the surface. In order to explore proton hole diffusion, we followed the positions of the individual PHCs in time and calculated the mean squared displacement projected onto the two primary surface crystal directions (±[12[combining macron]10] and ±[0001] for ZnO(101[combining macron]0), and ±[11[combining macron]00] and ±[0001] for ZnO(112[combining macron]0)). Fig. 7 shows the calculated mean squared displacements and some example trajectories of the PHCs.

At ZnO(101[combining macron]0), the calculated diffusion coefficient along the nonpolar direction [12[combining macron]10], 
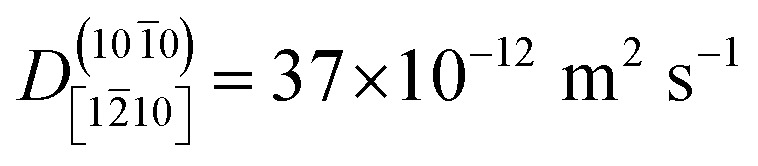
, is 20 times greater than the diffusion coefficient along the polar direction [0001], 
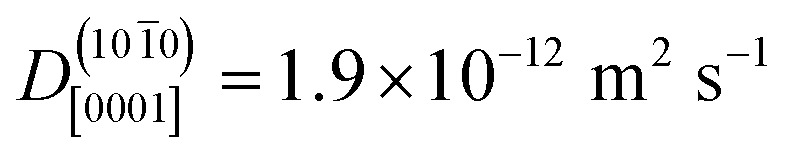
. Here, we note that the latter value is quite uncertain because of the small number of solvent-assisted PT events which contribute to PHC diffusion along [0001] (the small number of such events does not give rise to a “noisy” MSD, since the MSD is averaged over all PHCs); in fact, the biggest contribution to the calculated mean squared displacement along [0001], even for a correlation time of 25 ns (Fig. 7a), comes from individual surface-PT events, which do not contribute to PHC diffusion (see also Discussion). The calculated 
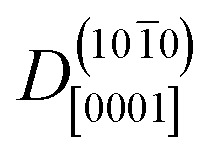
 is therefore likely overestimated.

At ZnO(112[combining macron]0), in contrast, diffusion along the *polar* directions is dominant: 
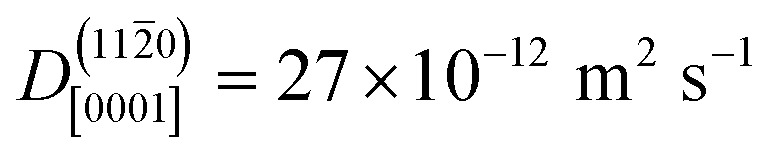
. However, the directionality is not as anisotropic as was the case for the other surface. Diffusion along the polar direction is only about 3 times faster than diffusion along the nonpolar direction: 
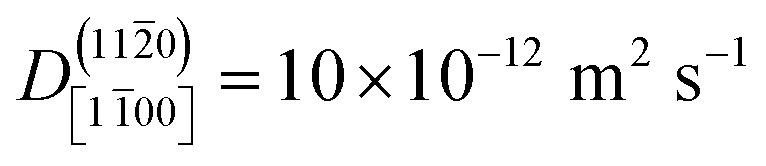
.

## Discussion

### Proton hole center diffusion network

The Grotthuss proton hole transport mechanism for OH^–^ in aqueous solution (Fig. 1) relies on the fact that an OH^–^ can participate in a proton transfer event with *multiple* (at least two) neighboring H_2_O molecules. If there were only one neighboring H_2_O molecule, the proton hole would have reached a “dead end”, forcing the next PT event to form again the previous OH^–^ (barring hydrogen bond fluctuations). In dilute aqueous solutions, OH^–^ accepts hydrogen bonds from three or four different H_2_O molecules,^2^ providing different pathways for the Grotthuss mechanism.

**Fig. 8:**
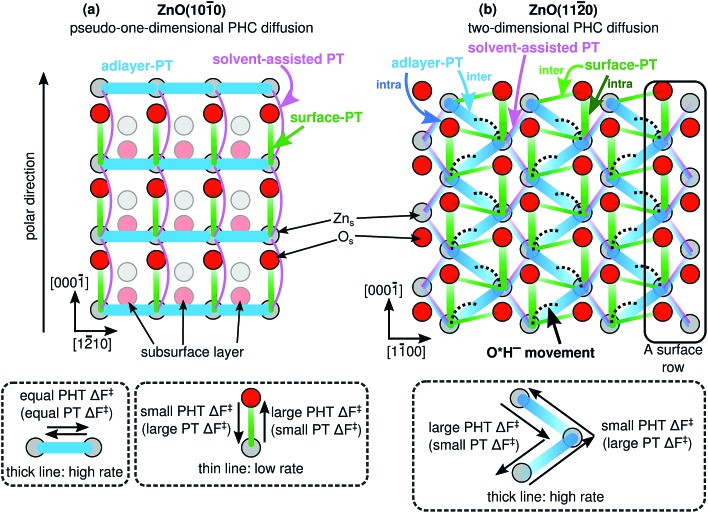
Calculated proton hole center (PHC) diffusion networks at the ZnO–liquid-water interface, for (a) the ZnO(101[combining macron]0) surface, and (b) the ZnO(112[combining macron]0) surface. The proton transfer (PT) and proton hole transfer (PHT) processes take place between PHCs, that can be located at surface O atoms, surface Zn atoms, or between two surface Zn atoms. Thicker solid lines indicate processes with greater rates. For lines with gradients, transferring the PHC from the dark end to the light end has a greater barrier than the reverse reaction. The dotted lines indicate movement of the PHC (Fig. 6), *i.e.*, a process without proton transfer.

Fig. 8 illustrates what we call the “proton hole center diffusion networks” at the ZnO(101[combining macron]0) and ZnO(112[combining macron]0) surfaces in contact with liquid water, by graphing all possible pathways that the PHC at any given position can take, for both proton transfer reactions (colored lines) and vehicular movement (black dotted lines). The thicknesses of the colored lines qualitatively indicate the rate of the individual PT processes, with thicker lines indicating greater rates. For lines with gradients, transferring the PHC from the dark end to the light end is associated with a larger barrier than the reverse reaction, as also explained in the dashed rectangles in Fig. 8. There is no PHC diffusion into the bulk liquid water, other than the transient species formed during solvent-assisted PT (which is not explicitly indicated in Fig. 8).

For example, at ZnO(101[combining macron]0), a PHC centered at Zn_s_^2+^ has five possible next locations: either of the two the neighboring Zn_s_^2+^ ions along the nonpolar ±[12[combining macron]10] direction (thick blue lines, corresponding to adlayer-PT events, *cf.*Fig. 4a), the neighboring O_s_^2–^ at greater coordinate along [0001[combining macron]] (green lines, corresponding to surface-PT events, *cf.*Fig. 4a), or either of the two neighboring Zn_s_^2+^ along ±[0001] (thin pink lines, corresponding to rare solvent-assisted PT events, *cf.*Fig. 5a). PHC(Zn_s_^2+^) thus constitutes a “hub” with several connections to neighboring surface ions, and can therefore potentially contribute to Grotthuss-like PHC diffusion at ZnO(101[combining macron]0). In contrast, PHC(O_s_^2–^) constitutes a “dead end” in the sense that a PHC centered at O_s_^2–^ only has *one* possible “next location”, namely the neighboring Zn_s_^2+^ with smaller coordinate along [0001[combining macron]]. If the network in Fig. 8a is considered as a mathematical graph, then the PHC(O_s_^2–^) vertices have degree 1. Thus, PHC(O_s_^2–^) cannot contribute to Grotthuss-like PHC diffusion at ZnO(101[combining macron]0).

Fig. 8a reveals the source of the very different calculated PHC diffusion coefficients (Fig. 7a) for diffusion along ±[12[combining macron]10] (horizontal) and ±[0001] (vertical) at ZnO(101[combining macron]0). Diffusion along ±[12[combining macron]10] is driven by consecutive adlayer-PT events (thick blue horizontal lines in Fig. 8a). Although the other major type of PT mechanism, surface-PT (green in Fig. 8a), transfers the PHC along the polar ±[0001] direction, it gives no long-range *diffusion* along ±[0001[combining macron]], since surface-PT transfers the PHC between Zn_s_^2+^ and O_s_^2–^ (eqn (3)), and PHC(O_s_^2–^) constitutes a dead end. Thus, diffusion along the polar ±[0001] direction can only happen *via* the solvent-assisted PT mechanism (Fig. 5), which we found to be very rare. The “jump” at around *t* = 1 ns for the pink PHC trajectory along [0001[combining macron]] in Fig. 7a corresponds to such a solvent-assisted PT event (depicted in Fig. 5a). However, for the vast majority of the time, the PHC positions simply oscillated between the O_s_^2–^ and Zn_s_^2+^ positions on neighboring surface “dimers” (see for example the green, blue, and orange trajectories along [0001[combining macron]] for ZnO(101[combining macron]0) in Fig. 7a). In contrast, along the nonpolar [12[combining macron]10] direction, several trajectories spanned several nm; in the examples in Fig. 7a, the PHCs indicated by the green and blue lines diffused about 1 nm (or about three lattice parameters along [12[combining macron]10]) in 2 ns.

We thus conclude that the *proton hole diffusion at the ZnO(101[combining macron]0)–water interface is pseudo-one-dimensional*, with a high rate of adlayer-PT events yielding diffusion along the nonpolar ±[12[combining macron]10] direction, and a very low rate of solvent-assisted PT events contributing to the relatively small diffusion along the polar [0001] direction.

At ZnO(112[combining macron]0), the proton hole center diffusion network (Fig. 8b) is in several ways different to what is the case at ZnO(101[combining macron]0). Firstly, PHC(O_s_^2–^) does not constitute a “dead end”, since there are two possible next locations of the PHC, corresponding to intra-surface-PT and inter-surface-PT events, respectively. Consequently, PHC(O_s_^2–^) can contribute to Grotthuss-like diffusion of the PHC. Moreover, the black dotted lines indicate the O*H^–^ movement from Fig. 6. However, vehicular O*H^–^ movement does not contribute to any long-scale diffusion of the PHC, since the bridging O*H^–^ species (Fig. 6) are very inactive for PT (ESI Fig. 4†).

The PT process at ZnO(112[combining macron]0) with the highest rate is inter-adlayer-PT (Fig. 4b), and Fig. 8b reveals that the concatenation of such PT events can yield a “zig-zag-like” diffusion of the PHC with net movement along the polar ±[0001] direction. For each individual inter-adlayer-PT event, the barrier to transfer the PHC from the dark end to the light end (downwards in the figure) is greater than the reverse process (see also Fig. 4). For the purpose of long-scale diffusion *via* the Grotthuss mechanism, *i.e.*, concatenated PT events, once a PHC has been transferred (for example one step downwards in the figure), there is an energy barrier associated with changing the proton transfer coordinate so that the next PT event along the same direction (as opposed to the opposite direction) can occur. For this reason, there is no perpetual energy gain from transferring the PHC along the polar direction. Instead, the amount of long-scale diffusion along both polar directions (up and down) are equivalent, since the system is in equilibrium.

Similarly, the PT processes with the second and third highest rates, namely intra-surface-PT and inter-surface-PT, can also be concatenated to yield net diffusion along the polar ±[0001] direction (“rectangular” diffusion along the green lines in Fig. 8b). Diffusion along ±[0001] thus occurs when the PHC alternately jumps back and forth between two surface rows. Net diffusion along the nonpolar ±[11[combining macron]00] directions occurs *via* intra-adlayer-PT events and solvent-assisted PT events (thin blue and pink lines in Fig. 8b). Such PT events transfer the PHC from one Zn_s_^2+^ to another Zn_s_^2+^ on the “other side” of the same surface row. Unlike the case at ZnO(101[combining macron]0), it is not only solvent-assisted PT events that enable PHC diffusion along the “minor” direction, but intra-adlayer-PT events also contribute to such diffusion. For this reason, it is not surprising that the one-dimensional diffusion coefficients at ZnO(112[combining macron]0) in both directions are much more similar in magnitude (differing only by a factor of 3, Fig. 7b) than what is the case at ZnO(101[combining macron]0).

Thus, although the PHC diffusion at ZnO(112[combining macron]0) is anisotropic, the similar magnitude of the one-dimensional diffusion coefficients lead us to the conclusion that *PHC diffusion at ZnO(112[combining macron]0) is two-dimensional*.

### Influence of pH

The proton hole diffusion coefficients, or equivalently, the proton diffusion coefficients, give a measure of the proton conductivity at the interface. In this work, the substrate (ZnO) is in contact with pure liquid water. This can be contrasted to previous simulation work exploring long-range proton and proton hole diffusion at solid/liquid interfaces (for example ref. 3, 10 and 11), where the diffusing species was explicitly added to the system. In such simulations, the substrate can interact with the diffusing species by influencing its preferred direction of diffusion, as well as aiding or inhibiting Grotthuss-like diffusion (as opposed to vehicular diffusion), but the surface has not “created” the diffusing species from reactions with the solvent. In the present work, we did not introduce any additional (“extrinsic”) protons or proton holes to the system, but instead followed the proton hole diffusion arising from the spontaneous dissociation and recombination of water on the surface. As a result, there is no net charge on the surface, and the present results for proton hole diffusion effectively concern ZnO surfaces at the point of zero charge (pzc), in the absence of specifically adsorbed counterions. The pzc of ZnO particles depends on the method of preparation, but is normally found at slightly basic pH, around pH 9.^46^ A higher pH would result in more deprotonation of O_s_H^–^ and O*H_2_ groups, and a lower pH in the protonation of O_s_^2–^ and O*H^–^ groups. Thus, the concentrations of species that contribute to proton conductivity at the interface depend on the pH. Adlayer-PT, which is the major contributor to PHC diffusion at both ZnO(101[combining macron]0) and ZnO(112[combining macron]0), requires that a mixture of O*H^–^ and O*H_2_ be present at the interface; thus, the pH may neither be too low nor too high for proton conduction to be efficient using the mechanisms presented in this work. Such pH-dependent differences in the physical behavior of the interface is of great interest for example for the application of ZnO-based materials as pH sensors.^15^ At low pH, other proton transfer mechanisms may become important; for example, proton conduction could potentially occur *via* O*H_3_^+^, which in our simulations at the pzc only transiently forms (ESI Fig. 2–5†). Characterizing the proton diffusion network at different pH values would serve as an excellent avenue for further exploration.

It is also possible for the surface to affect the pH of the surrounding solvent. The surface acidity can, in principle, be evaluated from the kinds of simulations that were performed in this work. For example, Wang *et al.*^41^ performed *ab initio* MD simulations for the GaN(101[combining macron]0)–water interface and calculated that surface to have p*K*_a_ = 3.0 ± 0.1, by considering the free-energy proton transfer barrier from the surface into the bulk liquid. However, in the present work, we did not observe the transfer of any PHCs, other than those during transient solvent-assisted PT events, into the bulk liquid. For this reason, we cannot explicitly evaluate the surface acidity from the present simulations.

### Implications for photocatalysis and nanomaterial design

The short-range and long-range proton transport properties of a surface are of fundamental importance to, for example, photoelectrochemistry and corrosion. In a recent first-principles simulation-based work, Wood *et al.*^42^ investigated short-range proton transfer reactions at the InP(001) and GaP(001) surfaces in contact with water, and provided an excellent discussion about how water diffusion and proton transfer reactivity determine the photoelectrochemical hydrogen evolution activity of these materials. In particular, the point on the surface at which a reactant (*e.g.*, H^+^ or H) forms need not be the same as the point at which it participates in the hydrogen evolution reaction.

ZnO and ZnO-derived materials have been shown to be promising materials for photocatalytic water splitting^16^,^17^ and photocatalytic degradation of polluting organic compounds in water.^18^ In the present study, we have shown that proton conduction on ZnO(101[combining macron]0) is pseudo-one-dimensional, whereas it is two-dimensional at ZnO(112[combining macron]0). This opens up interesting opportunities for the design of advanced ZnO-based nanomaterials in which such facet-dependent proton transport dimensionalities can be exploited. For example, our results would indicate that ZnO(112[combining macron]0), where proton diffusion is two-dimensional and comparatively fast, would allow for efficient, almost isotropic, proton diffusion from the place of the formation to the place of the reaction. In contrast, at ZnO(101[combining macron]0), the formation and reaction must take place at the same “coordinate” along the polar direction, thus limiting the probability that two reactants can meet to form the product. These results are particularly interesting in light of the fact that ZnO(101[combining macron]0) is a more stable surface than ZnO(112[combining macron]0).^20^

### Diffusion coefficients

Finally, we comment on the magnitudes of the calculated diffusion coefficients. Nuclear quantum effects (NQEs) are particularly pronounced for light elements like H, and have been shown to lower the proton transfer barriers of, for example, H_3_O^+^ and OH^–^ ions in aqeuous solution.^2^,^5^,^8^ Consequently, a consideration of NQEs leads to increased proton transfer rates and higher diffusion coefficients of these ions in water. It is not unreasonable to assume that similar considerations of NQEs would lead also to greater diffusion coefficients of the PHCs on the nonpolar ZnO surfaces. Although NQEs may affect the PT barriers, and, consequently, the PT rates and diffusion coefficients, they would not necessarily affect the relative diffusion coefficients along the polar and nonpolar crystal directions. An explicit treatment of NQEs lies outside the scope of the current work, but would give a more quantitatively accurate picture of the pertinent diffusion coefficients.

In simulations, the PT barriers and diffusion coefficients also depend on the underlying computational method.^6^ Here, we used a neural network potential fitted to reproduce a dispersion-corrected density-functional-theory-calculated potential energy surface. In a recent work, Chen *et al.*^9^ compared several different computational methods for the estimated diffusion coefficients of H_3_O^+^ and OH^–^ in aqueous solution, and found that a particular dispersion-corrected flavor of density functional theory overestimated the diffusion coefficients somewhat. Thus, although our present simulations on the one hand underestimate the diffusion coefficients because of the lack of nuclear quantum effects, they may well overestimate the diffusion coefficients as a result of the chosen reference method for the neural network parameterization, resulting in a partial cancellation of errors.

The one-dimensional proton hole diffusion coefficients in this work, for the crystal directions where the diffusion is significant, lie approximately in the range 10 to 37 × 10^–12^ m^2^ s^–1^ (Fig. 7). The room-temperature diffusion coefficients for the H_3_O^+^ and OH^–^ in bulk (3-D) water in dilute solution are 9.6 × 10^–9^ m^2^ s^–1^ and 5.4 × 10^–9^ m^2^ s^–1^, respectively. Because the diffusion of these ions in water is isotropic, the diffusion coefficients per spatial direction are a third of the quoted values, *i.e.*, 3.2 × 10^–9^ m^2^ s^–1^ and 1.8 × 10^–9^ m^2^ s^–1^, respectively. Thus, even the fastest (1-D) proton diffusion at one of the two ZnO–water interfaces in this work 
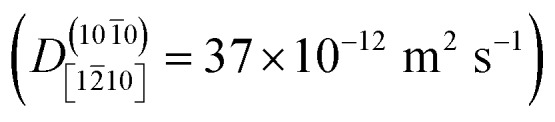
, is considerably slower than the diffusion of H_3_O^+^ and OH^–^ in water, amounting to only about 1–2% of the corresponding H_3_O^+^ and OH^–^ diffusion coefficients.

## Conclusions

The spontaneous dissociation of water near metal oxide interfaces, and the proton transport properties of the interface, are of fundamental interest for catalysis, electrochemistry, and geochemistry. Here, on the basis of large-scale molecular dynamics simulations employing a first-principles-quality neural network potential, we explored Grotthuss-like proton diffusion at the two most stable surfaces of zinc oxide in contact with liquid water, revealing fundamentally different surface properties for different surface geometries. In these simulations, no “extrinsic” protons or proton holes were introduced; instead, the protons diffuse *via* spontaneous water dissociation and recombination events at the interface. This is, to the best of our knowledge, the first time that such an analysis has been carried out for any metal-oxide–liquid-water interface. We found that, whereas proton diffusion at the ZnO(101[combining macron]0)–liquid-water interface is pseudo-one-dimensional, occurring mainly along the nonpolar ±[12[combining macron]10] directions, it is two-dimensional at the ZnO(112[combining macron]0)–liquid-water interface, occurring both along the nonpolar ±[11[combining macron]00] directions and the polar ±[0001] directions. For both surfaces, proton diffusion is considerably slower than the diffusion of H_3_O^+^ and OH^–^ in aqueous solution. These results highlight the importance of surface-specific properties of zinc oxide, with possible implications for applications of nanowires and nanoparticles, used for example as biosensors and photocatalysts.

## Methods

The potential energy surfaces of the ZnO–liquid-water interfaces were described by a high-dimensional neural network potential (NNP), that we previously developed and validated for the ZnO–liquid-water interface using training data for both the ZnO(101[combining macron]0) and ZnO(112[combining macron]0) surface in contact with liquid water.^34^ The NNP was parameterized to reproduce dispersion-corrected DFT-calculated energies and forces at the RPBE-D3 level of theory.^47^,^48^ RPBE-D3 has been shown to describe liquid water,^49^ as well as proton transfer reactions in NaOH solutions,^36^ very well.

The ZnO slabs were roughly 1 nm thick and had 48 Zn_s_ and 48 O_s_ surface atoms per side of the slab, and were separated by about 2.8 nm of liquid water (Fig. 2 and ESI†). The middle half of the ZnO slab was kept fixed during the simulations. The density of the liquid water was allowed to equilibrate in the NPT ensemble. The production simulations were run in the NVT ensemble for 44 ns using a timestep of 0.5 fs, following an equilibration period of 1 ns. The simulations were run using a custom module^49^ implemented in the LAMMPS program.^50^ Snapshots of the two systems are provided as ESI.†

Each H atom is assigned to be “covalently bound” to its nearest O atom. O atoms that do not belong to the ZnO crystal, but that are within 2.35 Å of a surface Zn ion, are considered to be adsorbed on the surface and are denoted with an asterisk.

We employed a method where we assign a “proton hole center”, PHC, to be located at each “free” O_s_^2–^ (surface O without any bound H), as well as at either the Zn or O position of O*H^–^. This assignment is useful because the number of surface atoms does not change during the simulation, whereas the number of water molecules adsorbed on the surface, which are the sources of the protons, fluctuates during the simulation as a result of exchange events with the liquid water film. Thus, in our two surface ZnO models of the ZnO(101[combining macron]0) and ZnO(112[combining macron]0) surfaces, there are 48 Zn_s_^2+^ and 48 O_s_^2–^ surface ions per side of the slab, and thus there is a total of 48 PHCs per side of the slab.

The location of a PHC is followed from one timestep to the next by minimizing the sum of squared distances moved by each PHC (obeying the minimum image convention):5
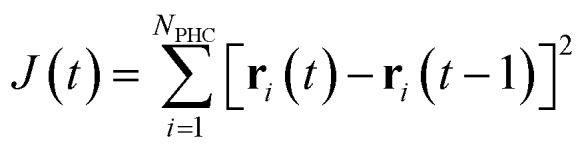

*i.e.*, we assign the 48 PHCs on one side of the slab such that the sum of squared distances to the positions of the PHCs in the preceding timestep is minimized. The minimal sum was found using the Hungarian method.^51^

By following the location of the PHCs, the mean squared displacement projected onto a direction *x* was calculated as

6 MSD_*x*_(*t*) = ) = 〈||*x*(*t*) – *x*(0)|^2^〉

where *x* is, for example, [0001[combining macron]], and where *x*(*t*) is the *x*-position of the PHC at time *t*, and the average , and the average 〈…〉 is taken over all time origins and all PHCs. The one-dimensional diffusion coefficient was then calculated as…, and the average 〈…〉 is taken over all time origins and all PHCs. The one-dimensional diffusion coefficient was then calculated as is taken over all time origins and all PHCs. The one-dimensional diffusion coefficient was then calculated as7
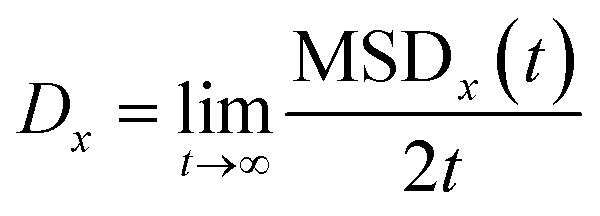
Here, we use a trajectory generated in a constant temperature simulation to calculate *D*_*x*_. The employed Nosé–Hoover thermostat can affect the dynamics of molecules, and our calculated diffusion coefficients should thus be seen as more qualitative than quantitative.

The proton-transfer free-energy landscapes (PTFELs) were calculated as follows: for each possible acceptor species (O_s_^2–^, O_s_H^–^, O*H^–^, O*H_2_, and solvent OH_2_), we scan through all of the donated hydrogen bonds, where a hydrogen bond O_d_H_d_···O_a_ exists if the distance *d*(O_d_–O_a_) < 3.5 Å and the angle ∠O_a_O_d_H_d_ < 30°.^52^ For each hydrogen bond, *δ* is calculated as *d*(H_d_···O_a_) – *d*(O_d_H_d_), where *d*(AB) is the distance between A and B. The donated hydrogen bond with the smallest value of *δ* is the one deemed “active” for PT, and the corresponding value of *δ* is called *δ*_min_. Depending on the nature and position of the active proton donor, the donor–acceptor pair is assigned to belong to a particular proton transfer coordinate (for example, “inter-adlayer-PT”; see ESI† for details). For each proton transfer coordinate, a histogram is created with a bin width of 0.1 Å, where the counter *W* is used for different intervals of *δ*_min_ in the simulation. The PTFEL is then constructed as

8 Δ*F*(*δ*_min_)/*k*_B_*T* = –ln(*W*(*δ*_min_))

Although *δ*_min_ ≥ 0. Å, for convenience the left hand sides of the PTFELs in Fig. 4 are plotted for the corresponding negative values of *δ*_min_.

## Conflicts of interest

There are no conflicts to declare.

## Supplementary Material

Supplementary information
