# Supplementary material for: One-dimensional vs. two-dimensional proton transport processes at solid–liquid zinc-oxide–water interfaces
Source: Chem Sci. 2018 Nov 5;10(4):1232–43. doi: 10.1039/c8sc03033b (PMC6349017; doi:10.1039/c8sc03033b)
Supplement: Supplementary file 1 [file SC-010-C8SC03033B-s001.pdf]

Electronic Supplementary Material (ESI) for Chemical Science.
This journal is © The Royal Society of Chemistry 2018

Supporting information for: One-dimensional vs. two-dimensional proton transport processes at solid–liquid zinc-oxide–water interfaces

Matti Hellström,[†,‡] Vanessa Quaranta,[†] and Jörg Behler*[,†,‡]

†*Lehrstuhl für Theoretische Chemie, Ruhr-Universität Bochum, 44780 Bochum, Germany*

‡*Universität Göttingen, Institut für Physikalische Chemie, Theoretische Chemie, Tammannstr. 6, 37077 Göttingen, Germany*

E-mail: joerg.behler@uni-goettingen.de

Supplementary method description

PT coordinate assignment

Below, we introduce the notation $O^{\sim}H_2$ and $O^{\sim}H^-$ to indicate "solvent" water molecules and hydroxide ions that are not adsorbed on the surface, i.e., molecules for which the distance to the nearest Zn^{2+} ion is greater than 2.35 Å. N.B. that with this definition, H_2O molecules that accept hydrogen bonds from O_sH^-, or that donate hydrogen bonds to O_s^{2-}, but which are not adsorbed on a surface Zn_s^{2+} ion, are considered to be solvent molecules. As in the main text, asterisks indicate oxygens adsorbed on the surface, and the subscript "s" is used to denote a ZnO surface atom.

The proton-transfer free-energy landscapes (PTFELs) were calculated as follows: Each H is assigned to be "covalently bound" to its nearest O atom. For each possible acceptor species (O_s^{2-}, O_sH^-, O^*H^-, $O^{\sim}H^-$, O^*H_2, $O^{\sim}H_2$), we scan through all of the accepted hydrogen bonds, where a hydrogen bond $O_dH_d \ldots O_a$ exists if the distance $d(O_d–O_a) < 3.5$ Å and the angle $\angle O_aO_dH_d < 30°$. For each hydrogen bond, δ is calculated as $d(H_d \ldots O_a) - d(O_dH_d)$, where $d(AB)$ is the distance between A and B. The donated hydrogen bond with the smallest value of δ is the one deemed "active" for PT, and the corresponding value of δ is called $\delta_{\min}$. Depending on the nature and position of the active proton donor, the donor-acceptor pair is assigned to belong to a particular proton transfer coordinate.

Below is a list of the PT reactions considered in this work. Each reaction is written in the form

$$\text{donor} + \text{acceptor} \rightleftharpoons \text{acceptor} + \text{donor} \tag{1}$$

where the left and right hand sides of the equation were assigned arbitrarily.

1. PT between surface and adlayer

   (a) $O_sH^- + O^*H^- \rightleftharpoons O_s^{2-} + O^*H_2$ **[surface-PT]**

   (b) $O_sH_2 + O^*H^- \rightleftharpoons O_sH^- + O^*H_2$

   (c) $O_sH^- + O^*H_2 \rightleftharpoons O_s^{2-} + O^*H_3^+$

   (d) $O^*H^- + O_s^{2-} \rightleftharpoons O^{*(2-)} + O_sH^-$

2. PT within adlayer

(a) $O^{*}H_2 + O^{*}H^- \rightleftharpoons O^{*}H^- + O^{*}H_2$ **[adlayer-PT]**

(b) $O^{*}H_3^+ + O^{*}H^- \rightleftharpoons O^{*}H_2 + O^{*}H_2$

3. PT involving the solvent

(a) $O_sH^- + O^{\sim}H^- \rightleftharpoons O_s^{2-} + O^{\sim}H_2$

(b) $O_sH^- + O^{\sim}H_2 \rightleftharpoons O_s^{2-} + O^{\sim}H_3^+$

(c) $O^{*}H_2 + O^{\sim}H^- \rightleftharpoons O^{*}H^- + O^{\sim}H_2$

(d) $O^{\sim}H_3^+ + O^{*}H^- \rightleftharpoons O^{\sim}H_2 + O^{*}H_2$

For ZnO($11\bar{2}0$), the surface-PT [1:(a)] and adlayer-PT [2:(a)] coordinates occurred for different nonequivalent placements of the donors and acceptors relative to each other and the surface. The algorithm for determining the coordinate of PT donor-acceptor pair $O_dH_d \ldots O_a$ for surface-PT and adlayer-PT at ZnO($11\bar{2}0$) was as follows:

- **surface-PT [1:(a)] at ZnO($\mathbf{11\bar{2}0}$)**

  $O_sH^- + O^{*}H^- \rightleftharpoons O_s^{2-} + O^{*}H_2$

  – If $O^{*}H^-$(LHS) or $O^{*}H_2$(RHS) coordinates two surface Zn ions (a "bridging" $O^{*}H^-$ or $O^{*}H_2$)

  $\rightarrow$ **1:(a):iv**

  – Otherwise, define the angle θ as the angle formed by three points projected onto the surface plane (i.e., three points with one coordinate along [$1\bar{1}00$] and one coordinate along [$000\bar{1}$]), see Supplementary Figure S1. The first point is the position of O_d, the second point the position of O_a, and the third point has the same coordinate along [$1\bar{1}00$] as O_a but a greater coordinate along [$000\bar{1}$].

    * $50° \leq \theta < 130° \rightarrow$ **1:(a):i [inter-surface-PT]**
    * $\theta < 50°$ (LHS) or $\theta > 130°$ (RHS) $\rightarrow$ **1:(a):ii [intra-surface-PT]**
    * $\theta > 130°$ (LHS) or $\theta < 50°$ (RHS) $\rightarrow$ **1:(a):iii** ["alternative" intra-surface-PT]

- **adlayer-PT [2:(a)] at ZnO($\mathbf{11\bar{2}0}$)**

O^*H_2+ O^*H^- $\rightleftharpoons$ O^*H^-+ O^*H_2 [distinction between LHS and RHS shown in Supplementary Figure S1]

Each surface row has a "left" and "right" side. Denote the average surface Zn coordinate along [11$\bar{0}$0] for row i as x_i^{left} and x_i^{right} for the left and right sides of the row, respectively (Supplementary Figure S1). Define θ as for surface-PT.

- If the x-coordinate of the transferred proton (H_d) lies between the left and right hand sides on adjacent rows, i.e., $x_i^{\text{right}} < x < x_{i+1}^{\text{left}}$

  → **2:(a):i [inter-adlayer-PT]**

  * $\theta < 90°$: LHS (O^*H_2 at greater [000$\bar{1}$]-coordinate than O^*H^-)
  * $\theta > 90°$: RHS (O^*H_2 at smaller [000$\bar{1}$]-coordinate than O^*H^-)

- If the x-coordinate of the transferred proton (H_d) lies between the left and right hand sides on the same row, i.e., $x_i^{\text{left}} < x < x_i^{\text{right}}$

  → **2:a:(ii) [intra-adlayer-PT]**

  * $\theta < 90°$: LHS (O^*H_2 at greater [000$\bar{1}$]-coordinate than O^*H^-)
  * $\theta > 90°$: RHS (O^*H_2 at smaller [000$\bar{1}$]-coordinate than O^*H^-)

For surface-PT, the LHS and RHS differ by the chemical species involved, and the angle θ is used to assign the PT donor-acceptor pair to one of the three different surface-PT coordinates (see the probability density function of θ in Supplementary Figure S1). In contrast, for adlayer-PT, θ is used to determine whether the PT pair is at the LHS or the RHS (Supplementary Figure S1), and the x-coordinate is used to assign the PT pair to one of the two adlayer-PT coordinates.

For each proton transfer coordinate, a histogram is created with a bin width of 0.1 Å, where the number of times W different ranges of δ_{min} occur in the simulation dump files. The PTFEL is then constructed as

$$\Delta F(\delta_{\text{min}})/k_{\text{B}}T = -\ln[W(\delta_{\text{min}})] \qquad (2)$$

Although $\delta_{\text{min}} \geq 0$ Å, for convenience the left hand sides of the PTFELs in the various figures are plotted for the corresponding negative values of δ_{min}.

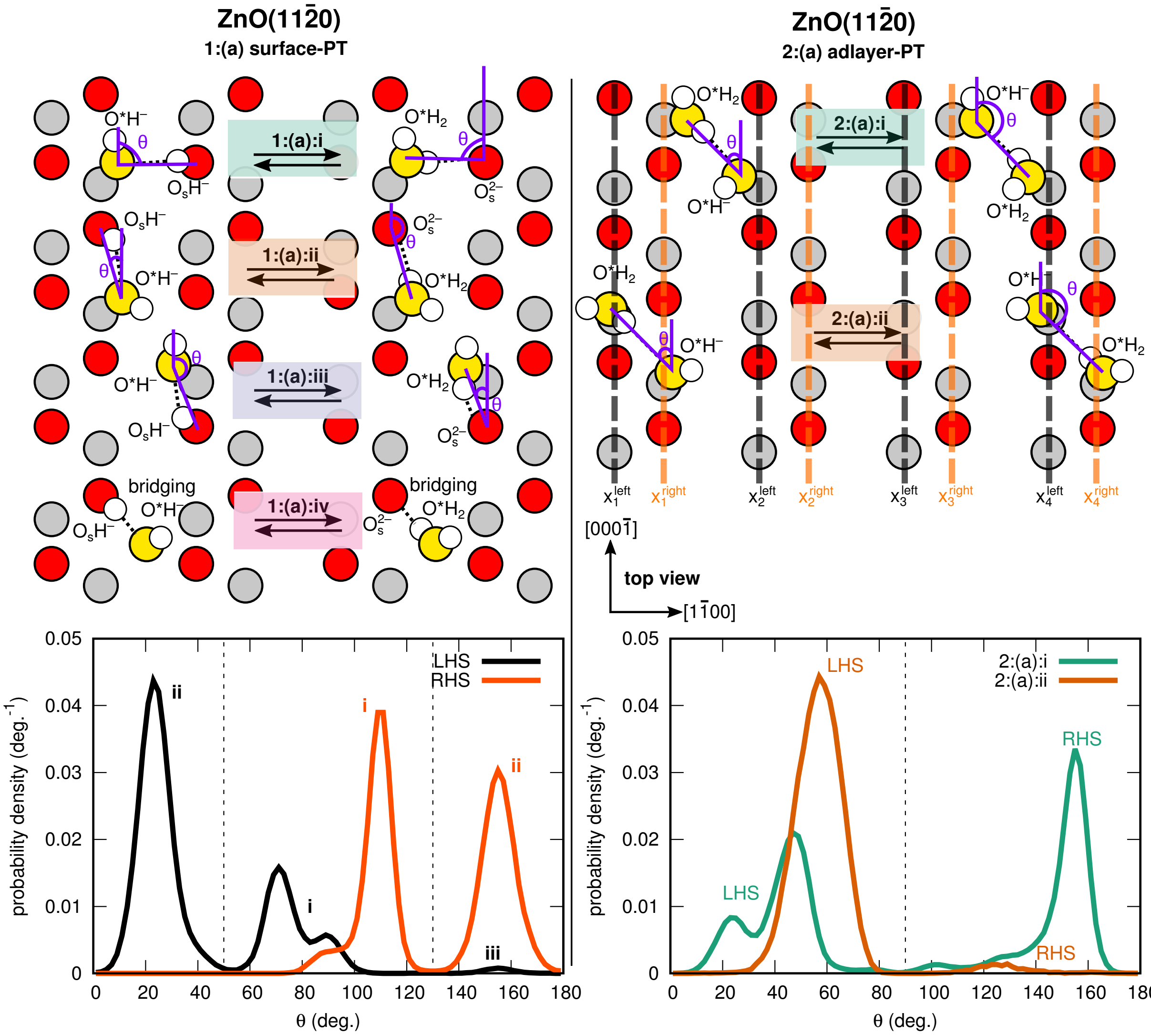

Supplementary Figure S1: Top: schematic pictures of the surface-PT and adlayer-PT coordinates at ZnO(11$\bar{2}$0), including illustrations of the angle θ. Bottom: Probability density functions of the angle θ. For ZnO(10$\bar{1}$0), the probability density function is calculated only for O^*H^- and O^*H_2 that coordinate 1 Zn_s^{2+} (i.e., reaction 1:(a):iv is excluded).

Proton hole centers vs. proton centers

We elected to follow the diffusion of proton holes, as opposed to the diffusion of protons, in our calculations of the mean squared displacement and diffusion coefficients D (see main text). It is, in principle, possible to apply an equivalent analysis for the diffusion of protons. However, this leads to rather counterintuitive definitions of "proton centers", since the number of adsorbed water molecules, which are the sources of the protons, fluctuates during the simulation as a result of exchange events with the liquid water film. Thus, not all surface Zn ions have a molecule adsorbed on them. Moreover, the bridging O^*H^- species at ZnO(11$\bar{2}$0) (see e.g. Supplementary Figure S1) essentially "occupies" *two* surface Zn ions, making them largely inaccessible for the adsorption of additional water molecules. However, one of the surface Zn ions can become accessible for additional water adsorption after the "O^*H^- movement" mechanism depicted in Figures 6 and 7 of the main text. In the calculation of the mean squared displacement, it is imperative that the number of proton (hole) centers remains constant, since otherwise the time correlation function would be undefined.

For completeness, Table S1 lists the definitions of the proton hole centers that we used in this work, together with definitions of "proton centers", defined so that the number does not fluctuate during the simulation, that we also could have used.

Table S1: Species that constitute proton hole centers and proton centers.

Proton hole centers	Proton centers
O_s^{2-}	${Zn_s}^{2+}$ (no adsorption)
O^*H^-	${Zn_s}^{2+}$ with adsorbed O^*H_2
	${Zn_s}^{2+} - O^*H^- - {Zn_s}^{2+}$ (O^*H^- bridging two ${Zn_s}^{2+}$)
	O_sH^-

Supplementary Results

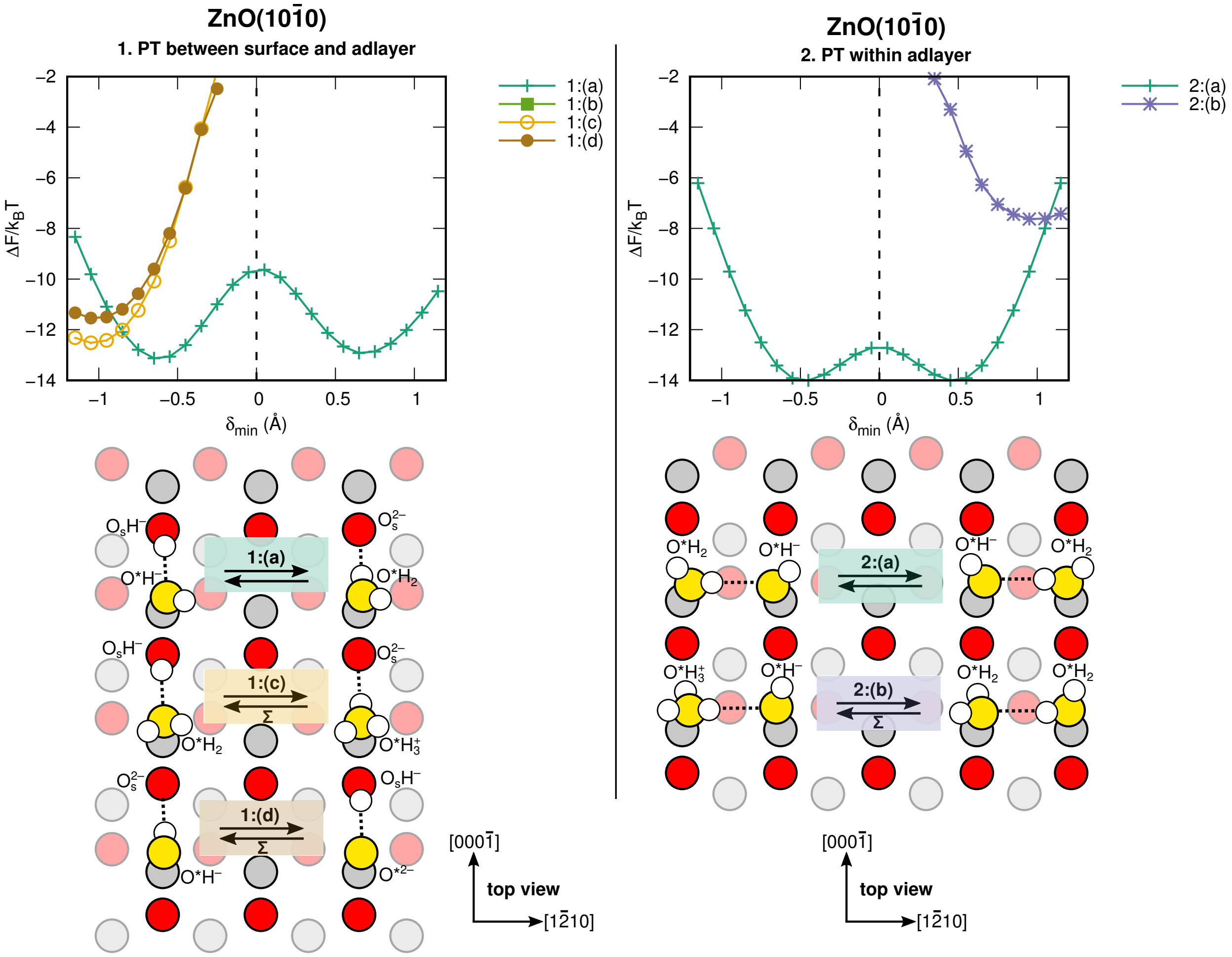

Supplementary Figure S2: Proton transfer free energy landscapes at ZnO(10$\bar{1}$0). Σ indicates that all possible geometrical arrangements of the donor-acceptor pairs are considered together. Neither the LHS nor the RHS of reaction 1:(b) form at ZnO(10$\bar{1}$0).

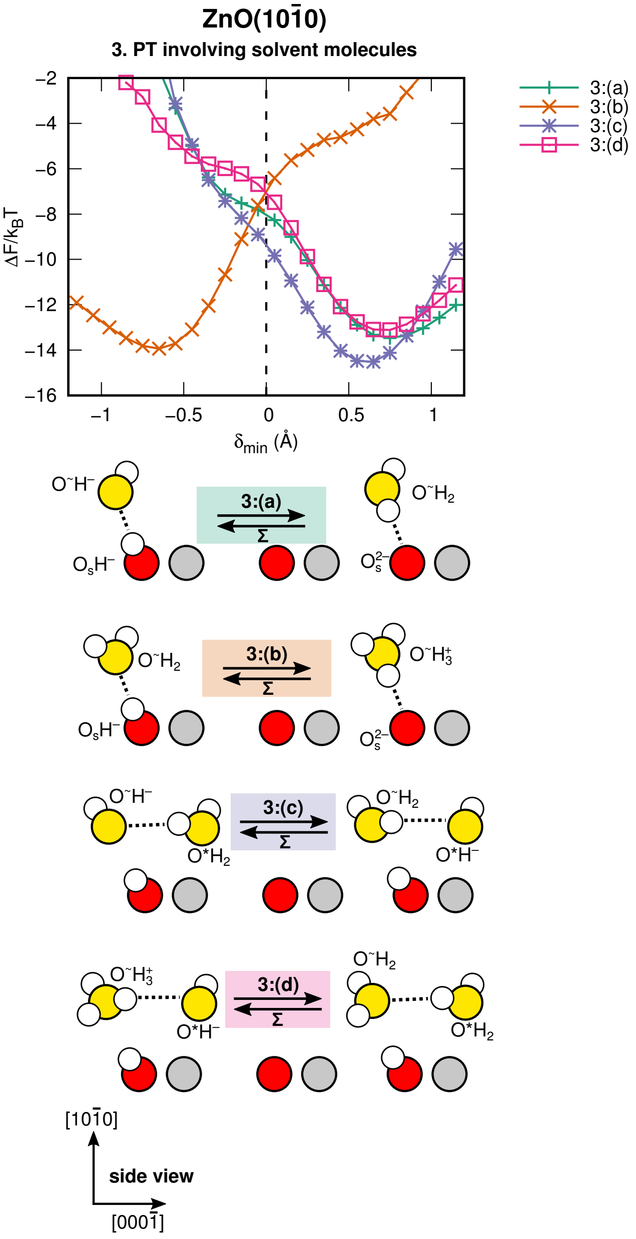

Supplementary Figure S3: Proton transfer free energy landscapes at ZnO(10$\bar{1}$0). Σ indicates that all possible geometrical arrangements of the donor-acceptor pairs are considered together.

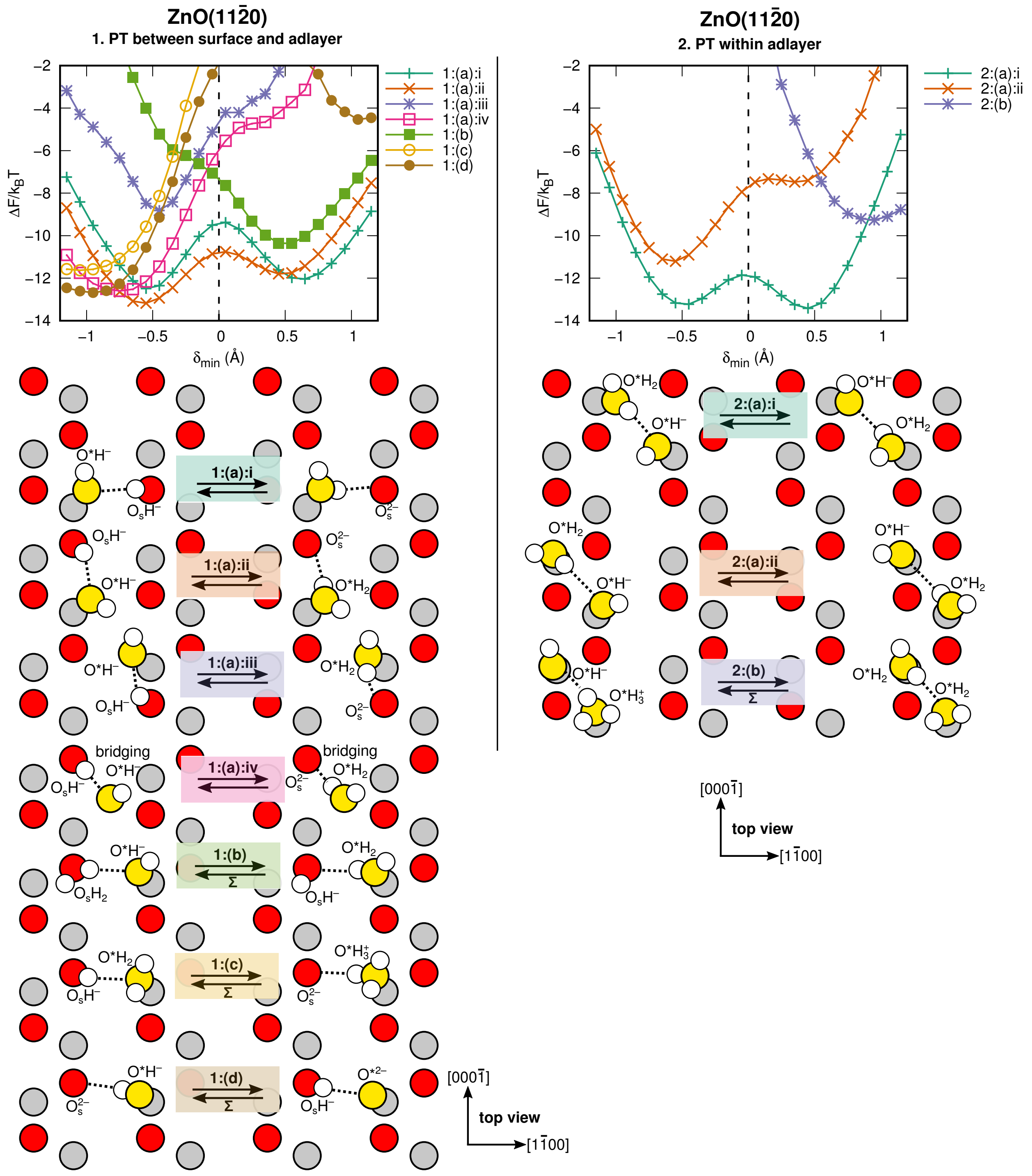

Supplementary Figure S4: Proton transfer free energy landscapes at ZnO(11$\bar{2}$0). Σ indicates that all possible geometrical arrangements of the donor-acceptor pairs are considered together (i.e., no distinction between "inter", "intra", etc., is made).

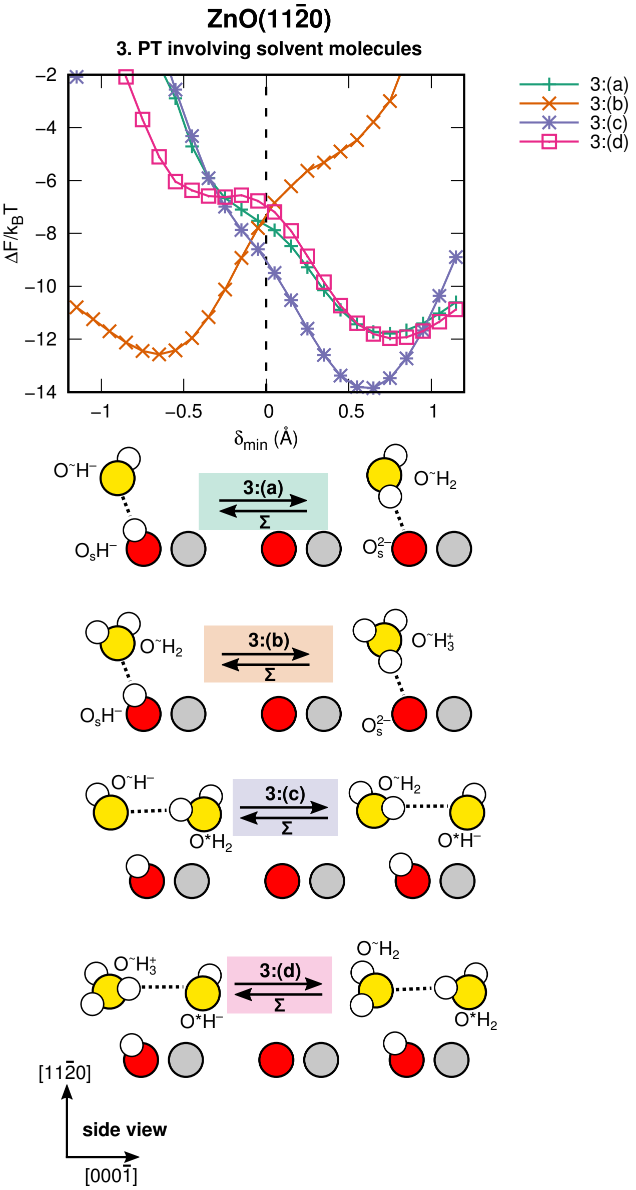

Supplementary Figure S5: Proton transfer free energy landscapes at ZnO(11$\bar{2}$0). Σ indicates that all possible geometrical arrangements of the donor-acceptor pairs are considered together (i.e., no distinction between "inter", "intra", etc., is made).

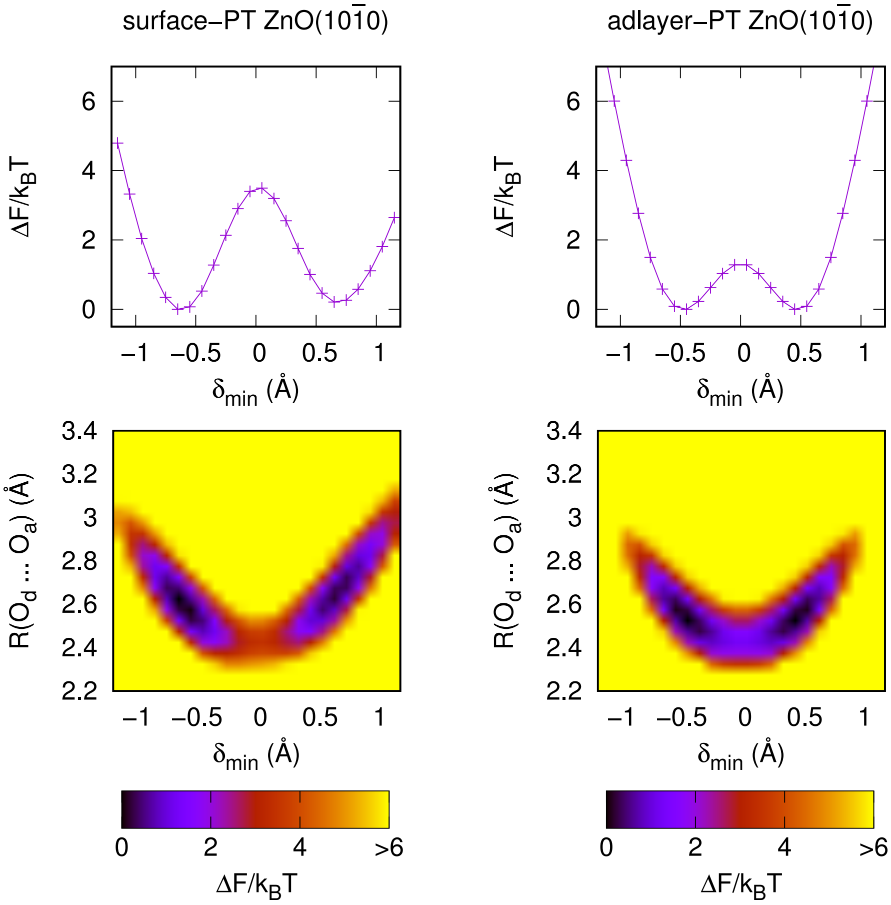

Supplementary Figure S6: Calculated one-dimensional and two-dimensional proton transfer free energy landscapes at the ZnO(10$\bar{1}$0) surface at $T = 300$ K. The curves have been shifted so that the free-energy minimum is at 0 $k_\mathrm{B}T$. In the two-dimensional cases, $R(O_d \cdots O_a)$ is the distance between the two O atoms.

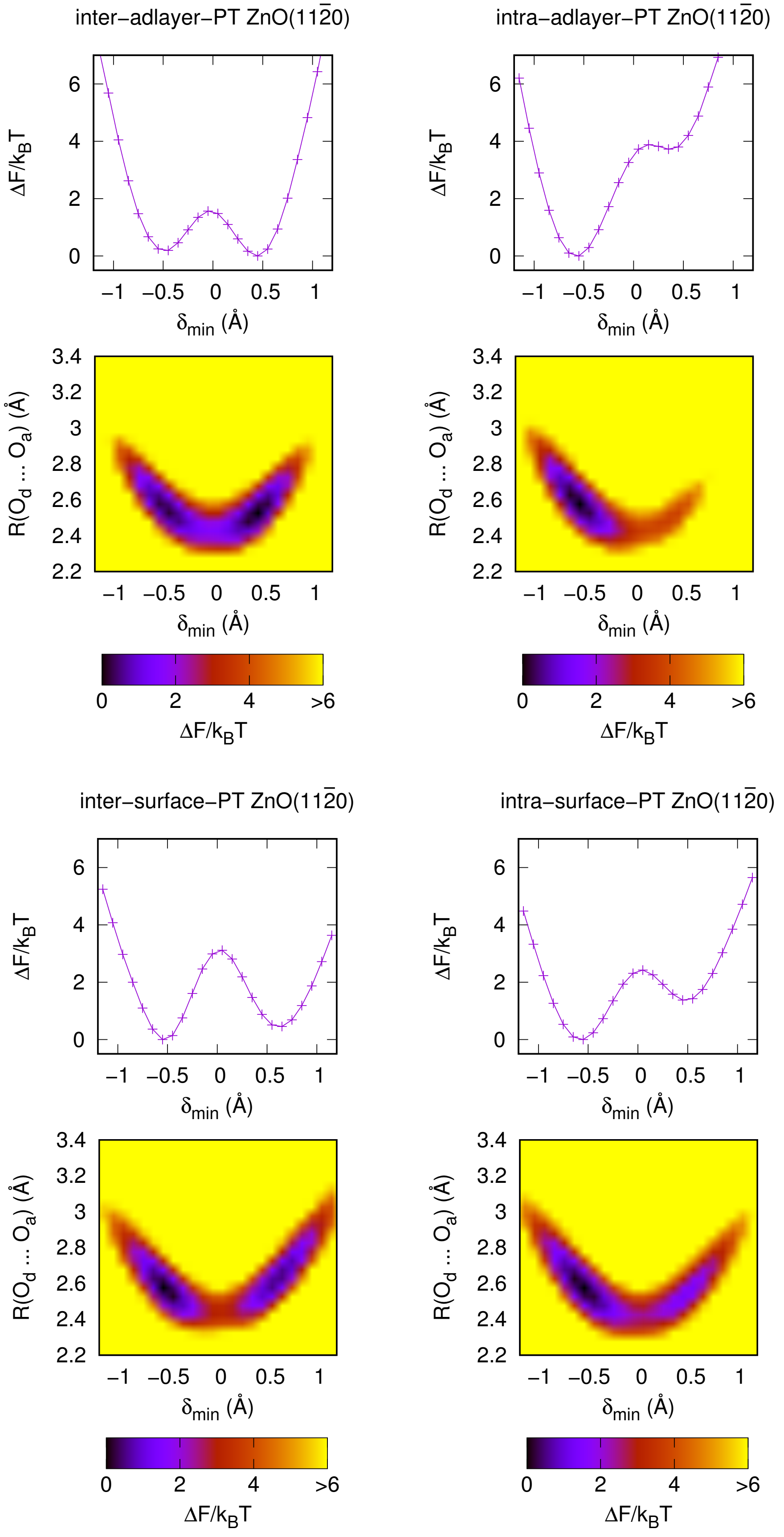

Supplementary Figure S7: Calculated one-dimensional and two-dimensional proton transfer free energy landscapes at the ZnO(11$\bar{2}$0) surface at $T = 300$ K. The curves have been shifted so that the free-energy minimum is at 0 $k_{\mathrm{B}}T$. In the two-dimensional cases, $R(O_d \cdots O_a)$ is the distance between the two O atoms.

Supplementary neural network validation

In the Supporting Information to Ref. S1, we presented the composition of the training and validation sets for both ZnO($10\bar{1}0$)–liquid-water and ZnO($11\bar{2}0$)–liquid-water interfaces. The training set contained a total of 15319 structures and the validation set contained 1712 structures. The root mean squared error (RMSE) for the total energies were 1.0 (1.2) meV per atom for the training (validation) sets, and the RMSEs for the atomic force components were 74 (76) meV/bohr. We additionally presented proton transfer free energy landscapes for the ZnO($10\bar{1}0$)–liquid-water interface calculated using both ab initio molecular dynamics (AIMD) and our parameterized neural network, and found good agreement between the two methods.

Here, we present supplementary validation of the neural network for the ZnO($11\bar{2}0$)–liquid-water interface. The model in Supplementary Figure S8a was used to initialize a neural network molecular dynamics simulation that was equilibrated in the NVT ensemble (T = 300 K) for 1 ns. The endpoint of this simulation was used to initialize an AIMD simulation using the RPBE-D3[S2,S3] functional (to which the neural network had been parameterized). Both the particle positions and velocities were used in the AIMD initialization. The AIMD simulation was 25 ps long and also run in the NVT ensemble (T = 300 K). For comparison, an identical 25 ps long simulation was run using the NN. The AIMD simulation was run using VASP[S4–S7] with Γ-point sampling, "hard" PAW potentials for O and H, and with an energy cutoff for the plane-wave basis set amounting to 700 eV. Those are the same settings that were used in the reference calculations for the neural network.

Supplementary Figure S8b shows the calculated PTFELs from an ab initio MD simulation and the corresponding neural network simulation. Because of the computational expense of the AIMD simulation, we were unable to obtain sufficient statistics to reliably distinguish between the different PT mechanisms (as done for the NN simulations in Figures S4 and S5). Instead, we simply compare the "total" proton transfer free energy landscapes. Supplementary Figure S8 shows that the agreement between the neural network and the ab initio molecular dynamics simulations is satisfactory, although the NN gives a somewhat smaller (by about 0.8 $k_{\mathrm{B}}T$) PT barrier.

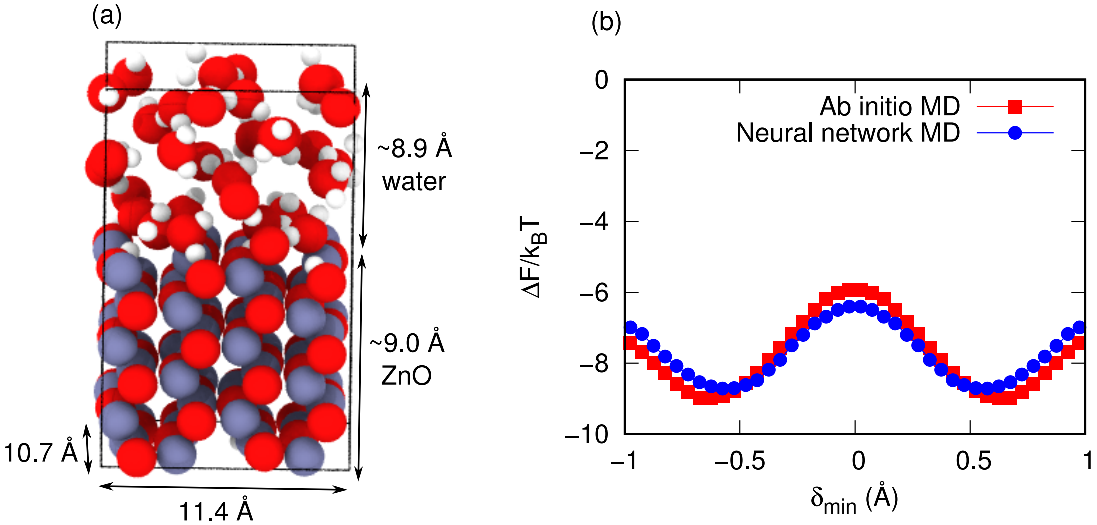

Supplementary Figure S8: (a) Structural model used for ab initio molecular dynamics and a corresponding neural network simulation. (b) Proton transfer free energy landscapes (PTFELs) calculated for the ZnO($11\bar{2}0$)–liquid-water interface. All proton transfer coordinates from Supplementary Figures S4 and S5 contribute to the PTFELs in this figure.

References

(S1) Quaranta, V.; Hellström, M.; Behler, J. *J. Phys. Chem. Lett.* **2017**, *8*, 1476–1483.

(S2) Hammer, B.; Hansen, L. B.; Nørskov, J. K. *Phys. Rev. B* **1999**, *59*, 7413–7421.

(S3) Grimme, S.; Antony, J.; Ehrlich, S.; Krieg, H. *J. Chem. Phys.* **2010**, *132*, 154104.

(S4) Kresse, G.; Hafner, J. *Phys. Rev. B* **1993**, *47*, 558.

(S5) Kresse, G.; Furthmüller, J. *Comput. Mater. Sci.* **1996**, *6*, 15.

(S6) Kresse, G.; Furthmüller, J. *Phys. Rev. B* **1996**, *54*, 11169.

(S7) Kresse, G.; Hafner, J. *Phys. Rev. B* **1994**, *49*, 14251.
